# Homozygous *CNP* Mutation and Neurodegeneration in Weimaraners: Myelin Abnormalities and Accumulation of Lipofuscin-like Inclusions

**DOI:** 10.3390/genes15020246

**Published:** 2024-02-15

**Authors:** Stefan H. Keller, Gary S. Johnson, Garrett Bullock, Tendai Mhlanga-Mutangadura, Malte Schwartz, Savannah G. Pattridge, Juyuan Guo, Gregg D. Kortz, Martin L. Katz

**Affiliations:** 1 Department of Veterinary Pathobiology, College of Veterinary Medicine, University of Missouri, Columbia, MO 65211, USA; shkgb6@missouri.edu (S.H.K.); gebkd2@mail.missouri.edu (G.B.); tendai@missouri.edu (T.M.-M.); sgpgqq@missouri.edu (S.G.P.); guoj@missouri.edu (J.G.); 2 Summit Veterinary Referral Center, Tacoma, WA 98409, USA; Mschwartz@summitvets.com; 3 VCA Sacramento Veterinary Referral Center, Sacramento, CA 95827, USA; gregg.kortz@vca.com; 4 Neurodegenerative Diseases Research Laboratory, Department of Ophthalmology, School of Medicine, University of Missouri, Columbia, MO 65212, USA

**Keywords:** neuronal ceroid lipofuscinosis, neurodegeneration, brain, optic nerve, cardiac muscle, mitochondria, lipofuscin

## Abstract

A progressive neurological disorder was observed in a male neutered Weimaraner. Clinical signs included fecal incontinence, lethargy, moderate paraparesis, proprioceptive pelvic limb ataxia, falling, cognitive decline, incoordination, decreased interest in food, changes in posture, and episodes of trance-like behavior. Neurologic signs were first observed at approximately 4 years, 10 months of age and progressed slowly. Magnetic resonance imaging showed generalized brain atrophy with areas of white matter pathology. Humane euthanasia was elected at 6 years, 7 months of age due to increasing severity of the neurological signs. Autofluorescent intracellular granules were observed in the cerebral and cerebellar cortexes, optic nerve, and cardiac muscle of the affected dog. These abnormal inclusions in the cerebral cortex and cardiac muscle immunolabeled with antibodies to mitochondrial ATP synthase subunit c protein, like that observed in the neuronal ceroid lipofuscinosis group of lysosomal storage diseases. Immunolabeling also demonstrated pronounced neuroinflammation in brain tissues. The ultrastructural appearances of the disease-related inclusion bodies in the brain and optic nerve were quite variable. The ultrastructure and locations of many of the inclusions in the nervous tissues suggested that they were derived, at least in part, from the myelin surrounding axons. The storage bodies in the cardiac muscle were located in mitochondria-rich regions and consisted of parallel arrays of membrane-like components interspersed with electron-dense flocculent material. The disease was characterized by pronounced abnormalities in the myelin of the brain and optic nerve consisting of distinctive areas of ballooning between the layers of myelin. The whole genome sequence generated from the affected dog contained a homozygous G-to-A missense mutation in *CNP*, which encodes proteins with CNPase enzyme activity and a structural role in myelin. The mutation predicts a Thr42Met amino acid sequence substitution. Genotyping of archived Weimaraner DNA samples identified an additional G > A variant homozygote with a clinical history and brain lesions similar to those of the proband. Of 304 Weimaraners and over 4000 other dogs of various breeds, the proband and the other Weimaraner that exhibited similar signs were the only two that were homozygous for the *CNP* missense variant. CNPase immunolabeling was widespread in brain tissues from normal dogs but was undetectable in the same tissues from the proband. Based on the clinical history, fluorescence and electron-microscopy, immunohistochemistry, and molecular genetic findings, the late-onset Weimaraner disorder likely results from the missense mutation that results in CNPase deficiency, leading to myelin abnormalities, accumulation of lysosomal storage bodies, and brain atrophy. Similar disorders have been associated with different *CNP* variants in Dalmatians and in human subjects.

## 1. Introduction

We recently reported a novel canine lysosomal storage disease (LSD) with similarities to the neuronal ceroid lipidoses in two Dalmatian littermates with a homozygous nullifying single-base deletion and reading-frame shift in *CNP*, the gene that encodes a protein with 2′,3′-cyclic-nucleotide 3′-phosphodiesterase enzyme activity (CNPase) and a structural role in myelin [1]. The affected littermates developed slowly progressing neurodegenerative signs that were first noticed when they were a year and a half old. The neurological signs included behavioral changes, cognitive decline, incoordination, and visual impairment. Results of magnetic resonance imaging of both littermates when 5 years old were consistent with diffuse cerebrocortical, cerebellar, and brainstem atrophy. After reaching eight years of age or older, some relatives of the littermates that were heterozygous for the *CNP* single-base deletion began showing neurologic signs including aggression, tremors, loss of appetite and weight, restlessness, behavioral changes, kyphosis, ataxia, sleep disturbance and loss of balance.

Due to the progression of neurodegenerative signs, the Dalmatian littermates with the homozygous deletion were euthanized when seven and eight years old. Fluorescence microscopic examination of unstained tissue from both littermates detected accumulations of cytoplasmic autofluorescent storage granules in the cerebellar cortex, cerebral cortex, optic nerve, and cardiac muscle. Immunohistochemical staining with antibodies raised to LAMP2 confirmed that the autofluorescence came from lysosome-derived inclusions. Electron microscopic examination of these same tissues found membrane-bounded cytoplasmic storage bodies with variable and complex ultrastructural appearances. In addition, the myelin sheaths surrounding the optic nerve axons exhibited abnormal ballooning between the layers of myelin membranes. Immunohistochemical staining with anti-CNPase antibodies produced pronounced staining in nerve fiber tracts of the cerebellum, cerebral cortex, optic nerve, and cardiac muscle from a control dog, but failed to detect CNPase antigen in the affected Dalmatian littermates [1].

We here describe a similar disorder that has occurred in a different dog breed, the Weimaraner. In addition, we report that the likely cause of the Weimaraner disease was a homozygous missense mutation in *CNP*.

## 2. Materials and Methods

**Figure 1:**
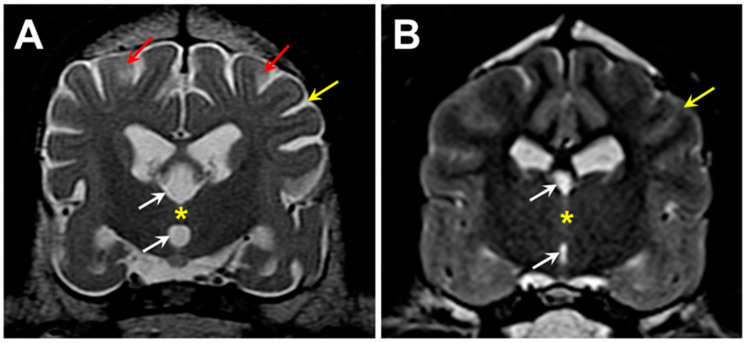
T2-weighted transverse MR images of the brain from the level of the interthalamic adhesion from the proband (**A**) and from an age- and weight-matched control diagnosed with idiopathic epilepsy (**B**). The proband exhibited cerebral parenchymal atrophy characterized by an abnormally small interthalamic adhesion (yellow asterisks), an enlarged third ventricle (white arrows), and widened subarachnoid spaces (yellow arrows). Areas of increased signal intensity within the cerebral cortex parenchyma white matter were also present (red arrows).

A 5-year, 1-month old neutered male Weimaraner (proband) was presented for neurological evaluation by a veterinary neurologist (MS) for an approximately 3-month history of pelvic limb ataxia, episodes of fecal incontinence, and lethargy. On neurologic examination, the proband was ambulatory with moderate paraparesis and proprioceptive pelvic limb ataxia characterized by a long-strided pelvic limb gait. A myelopathy was suspected. Standard MR images (1.5 Tesla instrument) of the thoracolumbar and cervical spine was performed, and no significant spinal cord abnormalities were identified. Additional MR imaging of the brain was performed (T2-weighted sagittal, dorsal, and transverse images; T1-weighted post-gadolinium contrast sagittal, dorsal, and transverse images; FLAIR and T2-weighted gradient echo transverse images), which revealed pronounced cerebral parenchymal atrophy (Figure 1). Cerebrospinal fluid analysis was unremarkable. Based on these findings, a neurodegenerative disease process was suspected. Approximately 1.5 years after the initial onset of clinical signs, humane euthanasia was elected due to the progression of neurologic dysfunction that included increased falling, worsening fecal incontinence, cognitive decline, incoordination, decreased interest in food, changes in posture, and episodes of trance-like behavior.

Following euthanasia, the eyes, brain and heart ventricular wall were collected and preserved, as described previously [2]. Chemically fixed slices of the retinas, optic nerves, cerebral cortex, cerebellar cortex, and cardiac muscle were cryo-embedded and sectioned with a cryostat. The unstained cryostat sections were examined for autofluorescence with a Zeiss Axiophot microscope (Carl Zeiss AG, Oberkochen, Germany) equipped with an Olympus DP72 color digital camera (Olympus Corp., Tokyo, Japan), as described previously [1]. Slices of fixed cerebral cortex, cerebellar cortex, and cardiac muscle were embedded in paraffin. Sections of the paraffin-embedded samples were immunostained using BioLegend (San Diego, CA, USA) anti-CNPase primary antibody (cat. no. 836403, dilution 1:500), Abcam (Cambridge, UK) anti-mitochondrial ATP synthase subunit c primary antibody (cat. no. ab180149, dilution 1:100), Abcam anti-ATPB primary antibody (cat. no. ab14730, dilution 1:100), Agilent Dako (Agilent Technologies, Santa Clara, CA, USA) anti-GFAP primary antibody (cat. no. Z0334, dilution 1:200), and Fujifilm Wako (Fujifilm North America, Lousiville, KY, USA) anti-Iba1 primary antibody (cat. no. 019-19741, dilution 1:100). Antigen retrieval and immunostaining was performed as described previously [1,3]. Additional slices of these tissues and of the optic nerve were examined with electron microscopy, as described previously [1].

Genomic DNA was prepared from EDTA-anticoagulated blood using a previously described procedure [4]. The proband’s DNA was submitted to the University of Missouri Genomics Technology Core Facility for library preparation and 2 × 150 bp paired-end sequencing on their Illumina NovaSeq 6000 sequencer. A previously described data-processing pipeline was used to align the sequence reads to a current canine reference genome assembly (Dog10K_Boxer_Tasha) and to analyze them with Ensembl annotation in conjunction with reads from 4024 other whole genome sequences obtained from the NCBI Sequence Read Archive (SRA) [1]. The SRA BioSample identifiers for all 4025 whole genome sequences used in this analysis are listed in Supplementary File S1. The amino acid positions for canine CNPase were numbered according to ENSCAFT00000102206.

We used an allelic discrimination assay to genotype individual dogs for a candidate variant at position 20,355,460 on chromosome 9. For this assay, the sequences of the PCR primers were 5′-CAGAGCTGCAGTTTCCTTTCCT-3′ and 5′-AGCGTCTTGCACTCTTGCA-3′. The competing probes sequences were 5′-VIC-TGGCCACCGTCTCCT-NFQ-3′ (reference allele) and 5′-FAM-TGGCCACCATCTTCCT-NFQ-3′ (variant allele).

## 3. Results

### 3.1. Microscopic Findings

**Figure 2:**
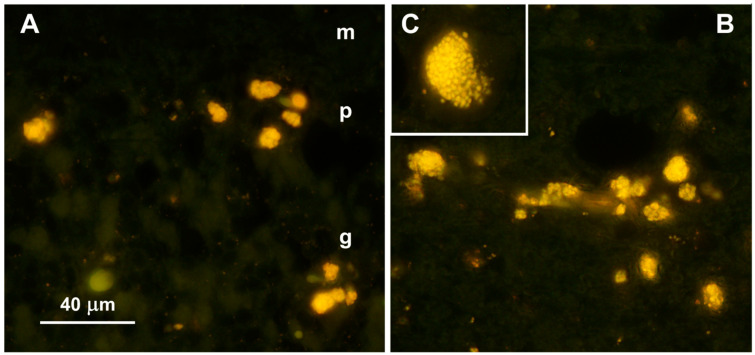
Fluorescence micrographs of unstained cryosections of the cerebellar cortex (**A**) and cerebral cortex (**B**,**C**) of the proband, showing yellow-emitting storage bodies in each tissue. In the cerebellar cortex, autofluorescent storage bodies were localized to the Purkinje cell (p) and granular (g) layers, with minimal autofluorescence in the molecular layer (m). In the cerebral cortex, cells containing the autofluorescent inclusions were distributed throughout the gray matter. In most of the affected cells, the storage bodies could be seen to consist of aggregates of autofluorescent granules (**C**). Bar in (**A**) indicates magnification of both (**A**,**B**).

**Figure 3:**
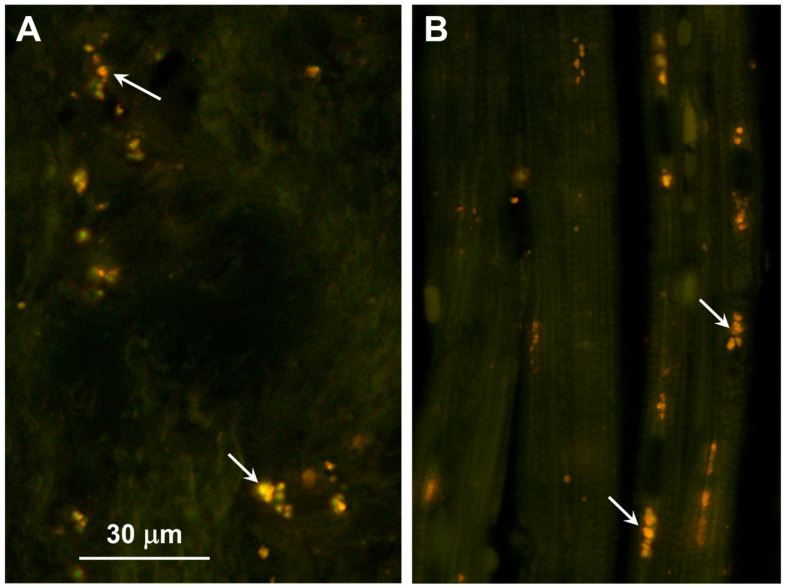
Fluorescence micrographs of unstained cryosections of the optic nerve (**A**) and cardiac muscle (**B**) of the proband showing autofluorescent storage bodies in each tissue (arrows). In the optic nerve, the storage bodies consisted primarily of individual small granules that had yellow to orange fluorescence emissions. In the cardiac muscles, the autofluorescent inclusions exhibited orange emission and were arrayed in linear groupings along the long axes of the muscle fibers. The bar in (**A**) indicates magnification of both micrographs.

Clusters of autofluorescent granules were present in cells of the cerebral cortex and cerebellar cortex of the proband (Figure 2). In the cerebellum, these inclusions were present primarily in the Purkinje cell and granule cell layers. In the cerebral cortex, the cells containing these inclusions were scattered throughout the gray matter. No inclusions with similar fluorescence properties were present in the neural retina, but autofluorescent granules were present in the optic nerve (Figure 3A). The granules in the optic nerve occurred either individually or as smaller clusters than seen in the brain tissues. Groups of autofluorescent inclusions were also present in the cardiac muscle, usually clustered in linear arrays within the muscle fibers (Figure 3B). The emission colors of the inclusions in these tissues ranged from golden yellow to orange, as detected by a color digital camera that was matched to the spectral sensitivity of the human eye.

**Figure 4:**
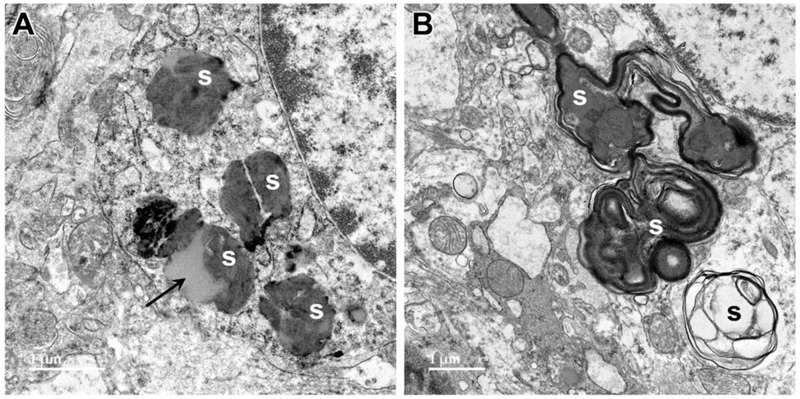
Electron micrographs showing examples of storage bodies (s) in cells of the cerebellar cortex of the proband. The storage bodies were heterogenous. The contents of some storage bodies were mixtures of electron-dense and lipid-like (arrow) components (**A**). The contents of other storage bodies consisted primarily of layers of membrane-like materials (**B**).

**Figure 5:**
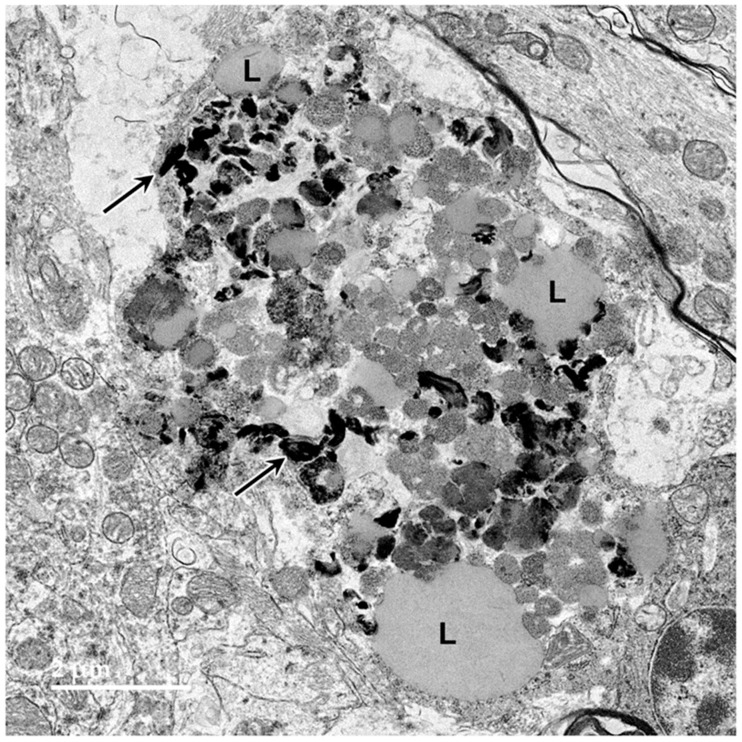
Electron micrograph of a large storage body in a cell of the cerebellar cortex of the proband. This type of storage body consisted of aggregates of large numbers of smaller components, some of which were quite electron-dense (arrows), and some of which had the appearance characteristic of lipid droplets (L).

**Figure 6:**
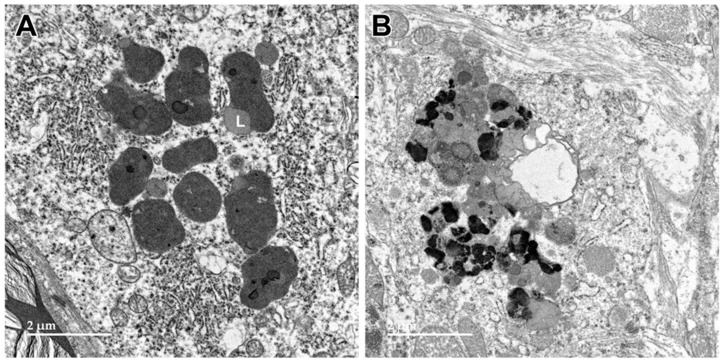
Electron micrographs of storage bodies in cells of the cerebral cortex gray matter from the proband ((**A**,**B**) are representative examples). Lipid-like components (L) were present in some of the storage bodies.

**Figure 7:**
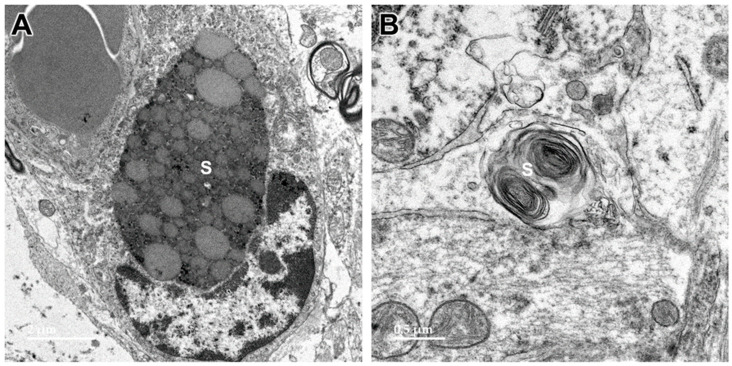
Electron micrographs showing additional examples of storage bodies (s) in cells of the cerebral cortex gray matter of the proband ((**A**,**B**) are representative examples).

**Figure 8:**
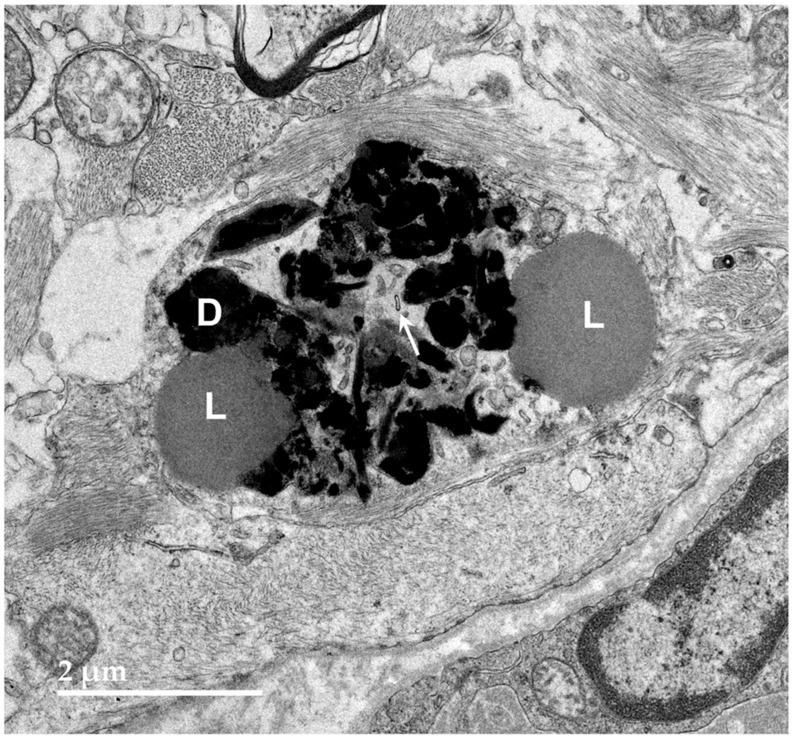
Electron micrograph of an intracellular inclusion in a cell of the cerebral cortical white matter of the proband. The contents of the inclusion body were heterogenous, consisting primarily of lipid-like components (L), aggregates of very electron-dense globular structures (D), and small vesicular structures (arrow).

**Figure 9:**
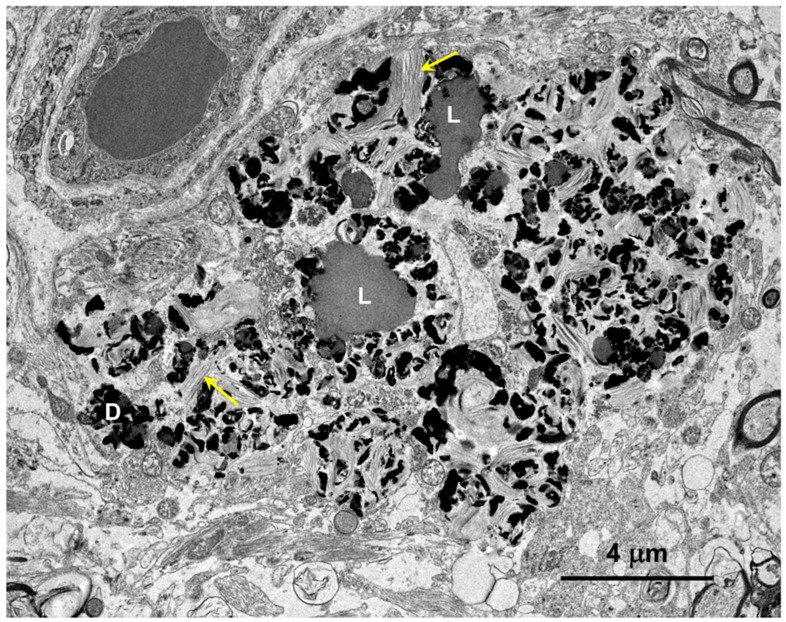
Electron micrograph of a large cluster of intracellular inclusions in the cerebral cortical white matter of the proband. The contents of the inclusion bodies within the cluster were heterogenous, consisting of lipid-like components (L), aggregates of very electron-dense globular structures (D), and membrane-like components (arrows).

**Figure 10:**
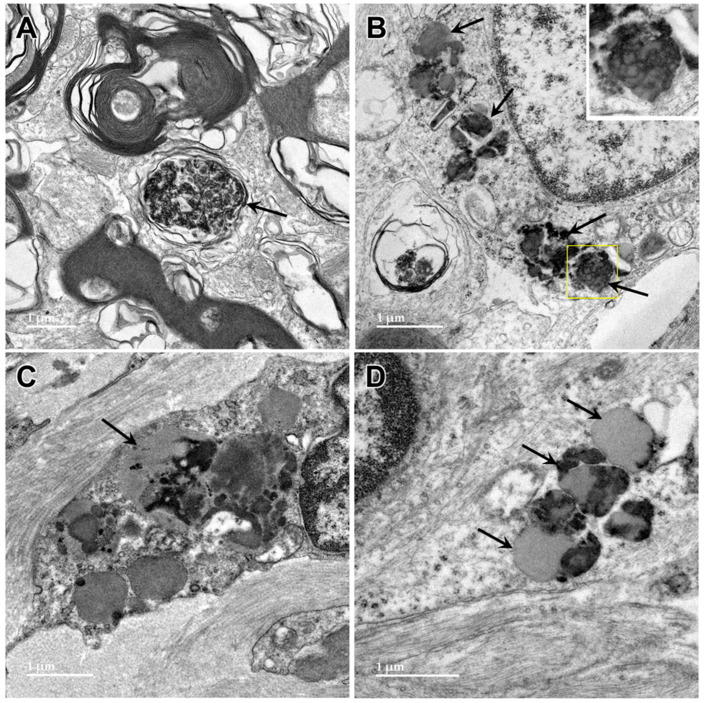
Electron micrographs showing lipofuscin-like inclusions in cells of the optic nerve from the proband (arrows). The inclusion framed in yellow in (**B**) is shown at higher magnification in the inset. The contents of these inclusions were quite heterogeneous and included lipid-like components, vesicular structures and irregularly shaped areas of high electron density. (**A**–**D**) illustrate the heterogeneity of the ultrastructure of the inclusions.

**Figure 11:**
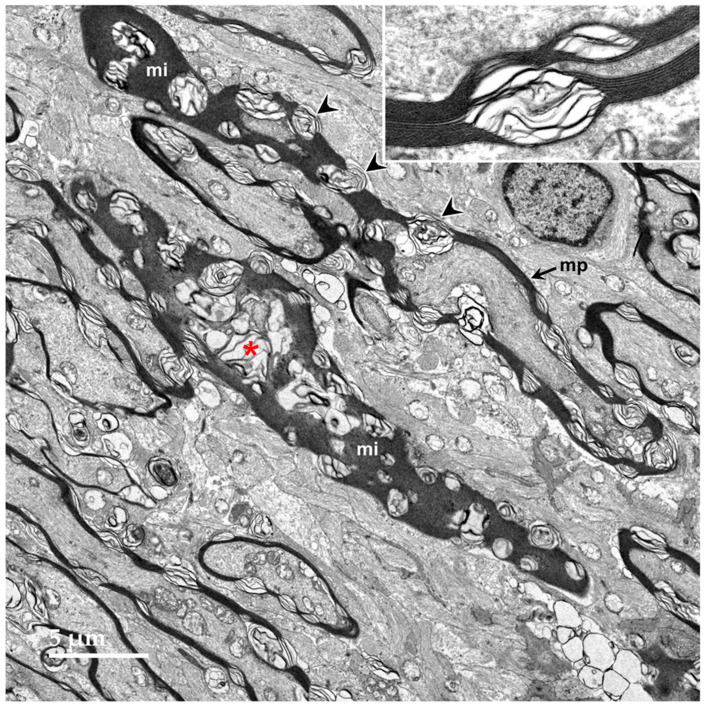
Oblique longitudinal section of the optic nerve from the proband. In some areas, the myelin sheaths surrounding the axons are seen in profile where the plane of section was perpendicular to the axonal membranes (mp), and in other areas, the plane of section near parallel with the myelin membranes (mi). In both orientations, areas of ballooning of the myelin sheaths could be seen (arrowheads and red asterisk). Inset shows at higher magnification where an area of tightly packed myelin membranes transitions to an area where they are ballooned apart.

**Figure 12:**
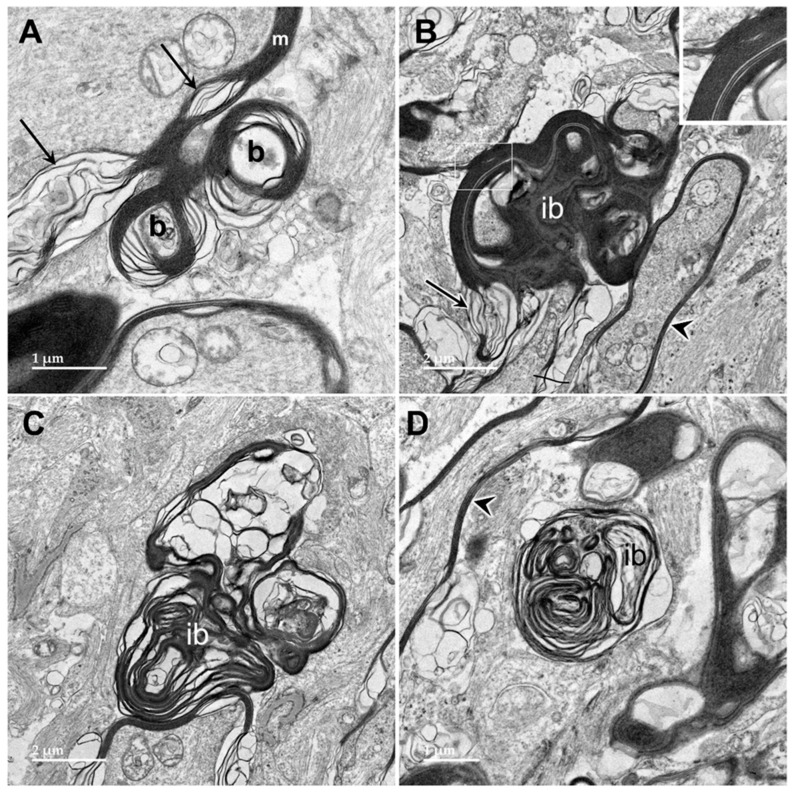
Electron micrographs of longitudinal sections of the optic nerve from the proband. The myelin sheaths surrounding the axons (m in (**A**), arrowheads in (**B**,**D**)) consisted of areas where the layers of membranes were tightly packed interspersed with regions of pronounced ballooning between the layers (arrows in (**A**,**B**)). In places, spherical buds projected out from the myelin sheaths (b in (**A**)). In other places, these buds were quite large (**B**) with the myelin folding back on itself to form large inclusion bodies (ib). Within these inclusions, some of the myelin-derived material retained the tight packing of normal myelin (inset in (**B**)). In other inclusion bodies, apparently derived from the myelin but separated from the sheaths, the membranous contents were more loosely packed and often formed fingerprint-like patters (**C**,**D**).

**Figure 13:**
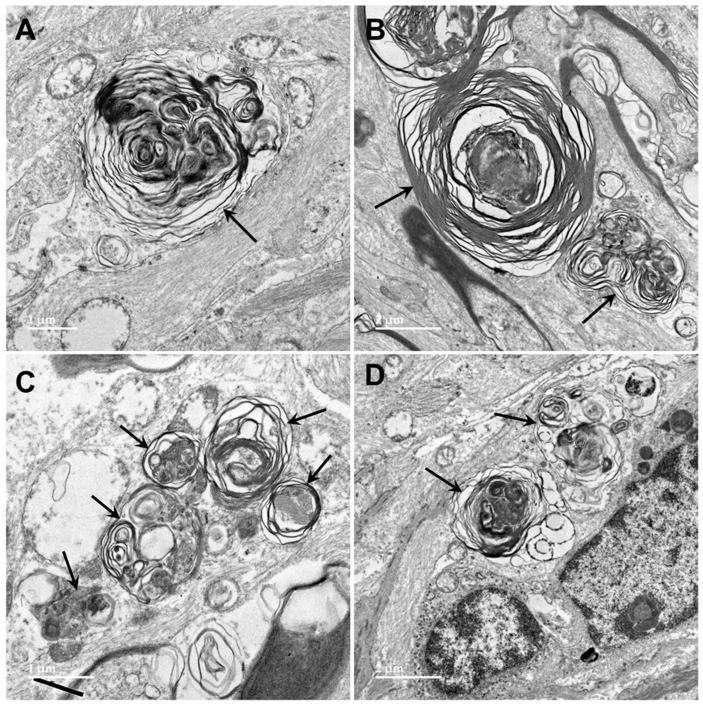
Electron micrographs of longitudinal sections of the optic nerve from the proband. Inclusion bodies (arrows) in cells of the optic nerve had ultrastructural features suggesting that they were derived from myelin, but also other features, including amorphous and flocculent electron-dense materials. (**A**–**D**) show representative examples of structures that appear to be derived from myelin membranes.

**Figure 14:**
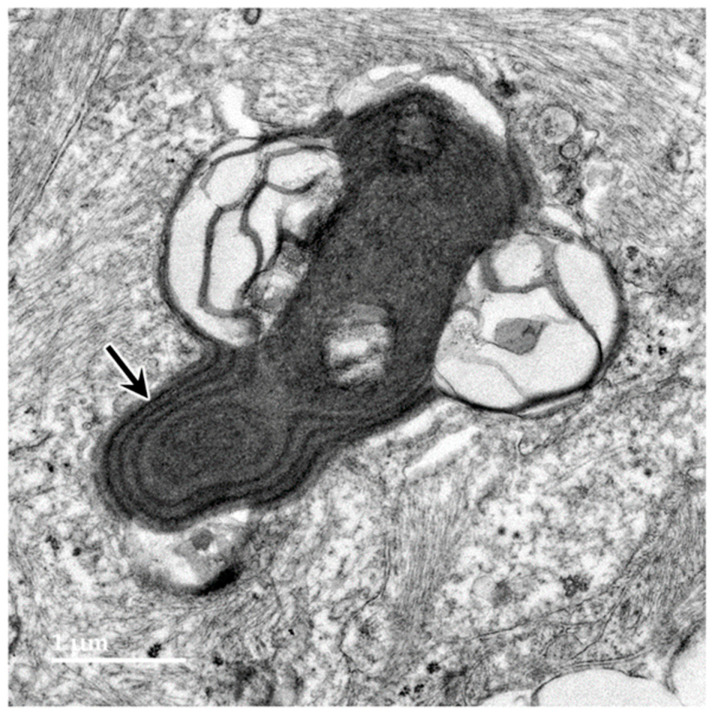
Electron micrograph of an inclusion body in an optic nerve neuron showing myelin-like membranes embedded in an electron-dense amorphous matrix (arrow) extending into electron lucent areas where the membranes are more loosely packed and irregularly arranged.

**Figure 15:**
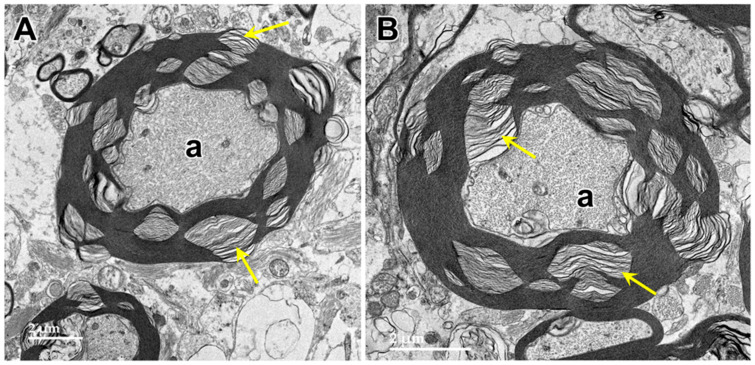
Electron micrographs of cross-sections of axons (a) in the cerebrocortical white matter of the proband. The myelin sheaths surrounding almost every axon contained numerous areas where there were pronounced gaps between the individual myelin layers (arrows). (**A**,**B**) are representative examples.

**Figure 16:**
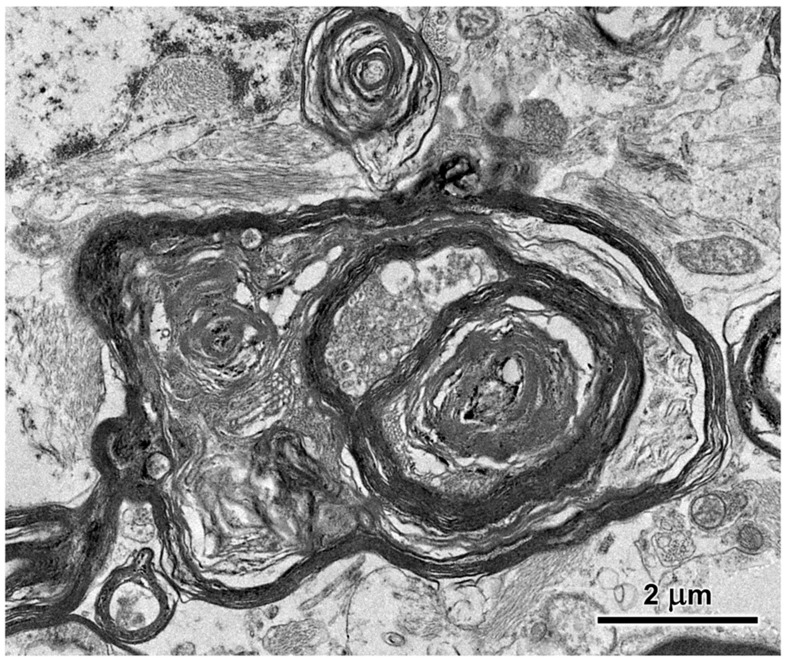
Electron micrograph of cross-section of a degenerating axon in the cerbrocortical white matter of the proband. The axoplasm has been largely replaced by myelin membranes that appear to have collapsed inward into the axon.

Electron microscopic examination of the cerebral cortex gray matter revealed the presence of membrane-bounded organelles containing mixtures of materials that included granular components of varying electron density, membranous components, and lipid-like inclusions (Figure 4 and Figure 5). Organelles with similar heterogeneous contents were present in the cerebellar cortex gray matter (Figure 6 and Figure 7), in the cerebral cortical and cerebellar white matter tracts (examples from the cerebral cortical white matter shown in Figure 8 and Figure 9), and in the optic nerve (Figure 10). The myelin sheaths of the optic nerve contained numerous areas of large abnormal gaps between the layers of myelin (Figure 11). In the optic nerve, it appeared that some of abnormal myelin had budded off from the axonal sheaths and was taken up into the adjacent cells forming structures that contained whorls of membrane (Figure 12, Figure 13 and Figure 14). In the brain white matter tracts the myelin sheaths surrounding almost all of axons exhibited distinctive gaps between the myelin layers (Figure 15). Some of the axons appeared degenerate, with the axoplasm replaced by myelin-derived membrane and membrane fragments that appeared to have collapsed inward into the axon (Figure 16).

**Figure 17:**
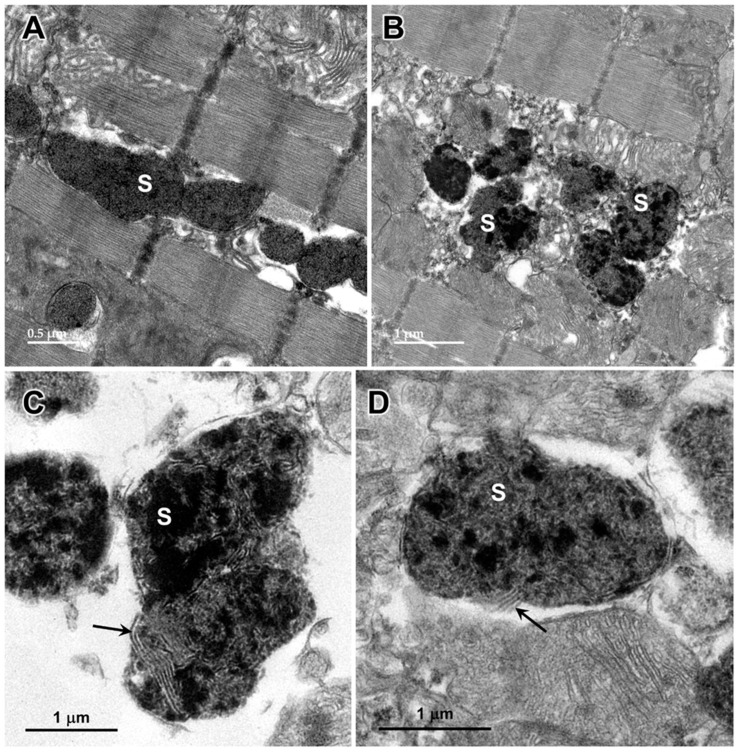
Electron micrographs showing storage bodies (s) in cardiac muscle from the proband. The storage bodies occurred in clusters between the myofibrils (**A**,**B**). At high magnification (**C**,**D**), the contents of the storage bodies could be seen to consist of parallel arrays of membrane-like structures (arrows) and clumps of very electron-dense amorphous materials.

Aggregates of abnormal electron-dense inclusion bodies were also present in the cardiac muscle fibers of the proband (Figure 17). These inclusions were localized to the mitochondria-rich regions of the cardiac muscle fibers. They were much more uniform in appearance than the inclusion bodies observed in the brain and optic nerve, consisting of parallel arrays of membrane-like constituents overlayed with patches of amorphous very electron-dense material (Figure 17C,D).

**Figure 18:**
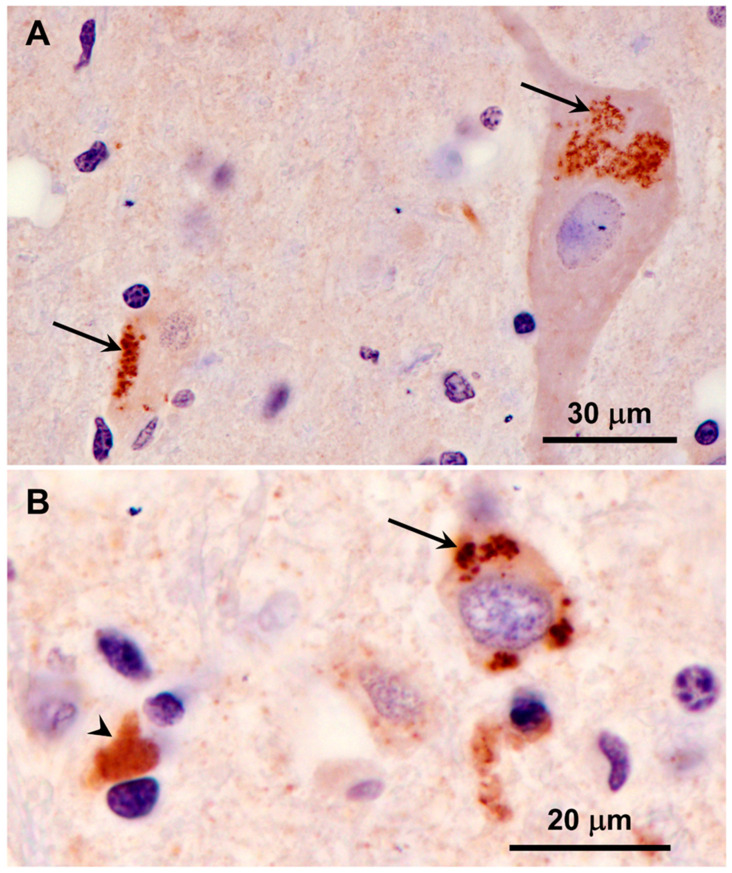
Immunohistochemical localization of mitochondrial ATP synthase subunit c protein in sections of cerebral cortex gray matter from the proband. Aggregates of punctate immunostained inclusions were present in large neurons (arrows in (**A**)), as well as smaller cells (arrow in (**B**)). In some of the smaller cells areas of more diffuse immunostaining was observed (arrowhead in (**B**)).

**Figure 19:**
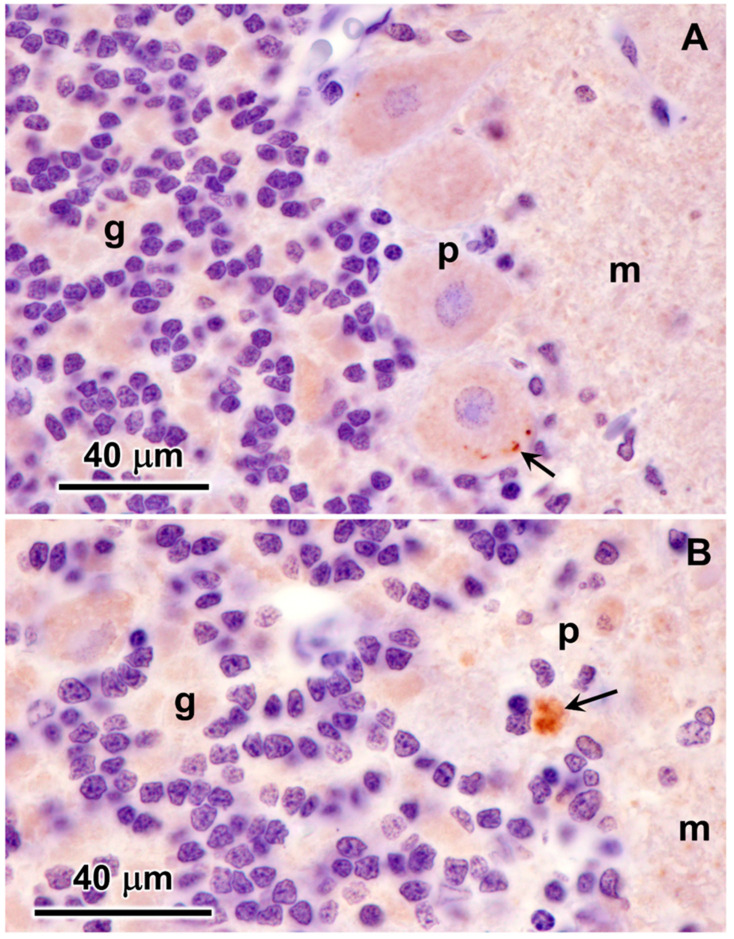
Immunohistochemical localization of mitochondrial ATP synthase subunit c protein in sections of cerebellar cortex from the proband. A small subset of Purkinje cells contained a few punctate immunostained inclusions (arrow in (**A**)). In addition, some small cells at the boundary between the granule cell and the Purkinje cell layers contained immunolabled inclusions (arrow in (**B**)). These cells were relatively rare. Layers of the cerebellar cortex: granular layer (g); Purkinje cell layer (p); molecular layer (m).

**Figure 20:**
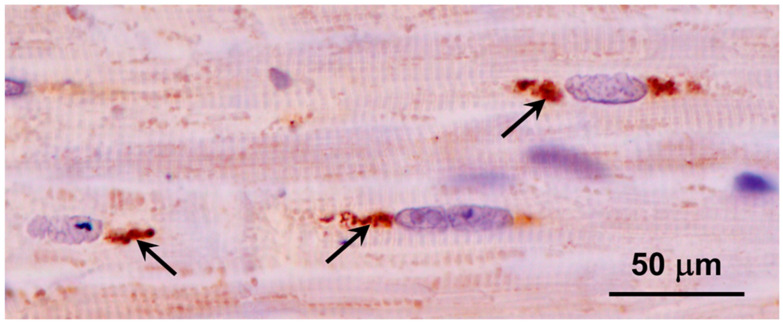
Immunohistochemical localization of mitochondrial ATP synthase subunit c protein in sections of cardiac muscle from the proband. Aggregates of immunostained inclusions were present in the muscle fibers adjacent to the cell nuclei (arrows).

A large fraction of the disease-related inclusions in the cerebral cortical gray matter were labeled with an antibody directed against the subunit c protein of mitochondrial ATP synthase (Figure 18). In contrast, only a very small subset of the disease-related inclusions in the cerebellar cortex were labeled with this antibody (Figure 19). In cardiac muscle, the disease-related inclusions exhibited strong immunolabeling with the subunit c antibody (Figure 20). None of the disease-related inclusions in either brain tissue or cardiac muscle exhibited labelling with an antibody that binds the β subunit of mitochondrial synthase.

**Figure 21:**
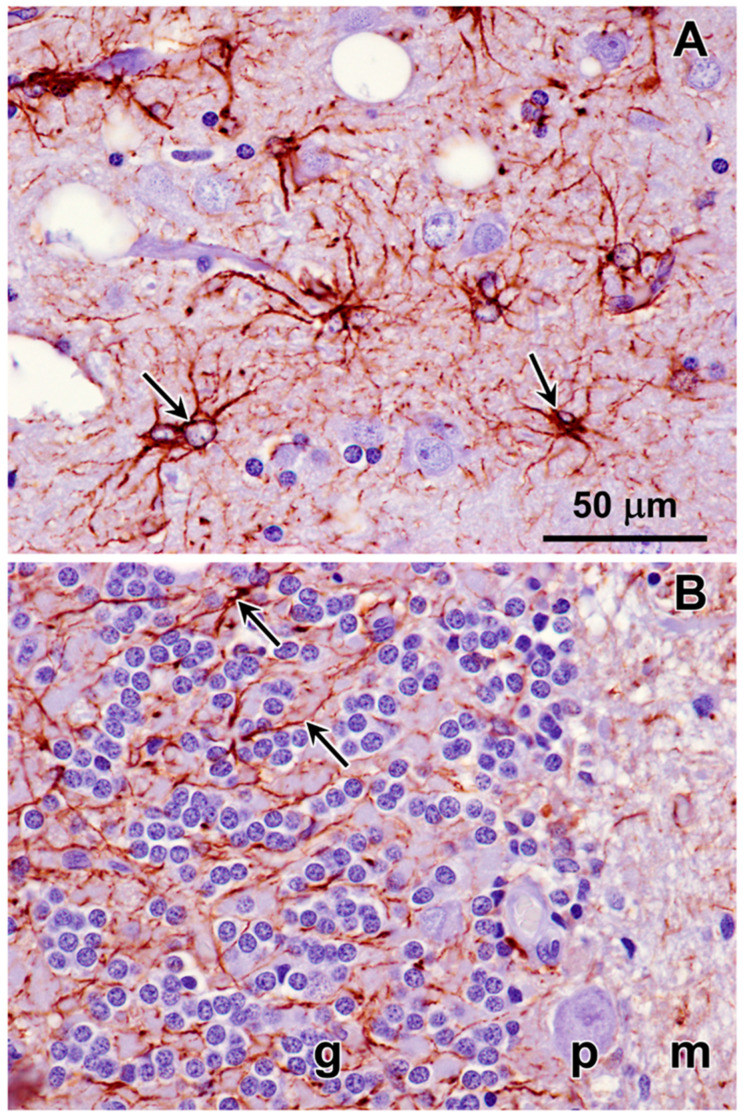
Immunohistochemical localization of the activated astrocyte marker GFAP in sections of cerebral cortex (**A**) and cerebellum (**B**) from the proband. Activated astrocytes were abundant throughout the cerebral cortex gray matter (arrows in (**A**)). In the cerebellum, labeled processes of activated astrocytes were abundant primarily in the granular layer (arrows in (**B**)). Layers of the cerebellar cortex: granular layer (g); Purkinje cell layer (p); molecular layer (m).

**Figure 22:**
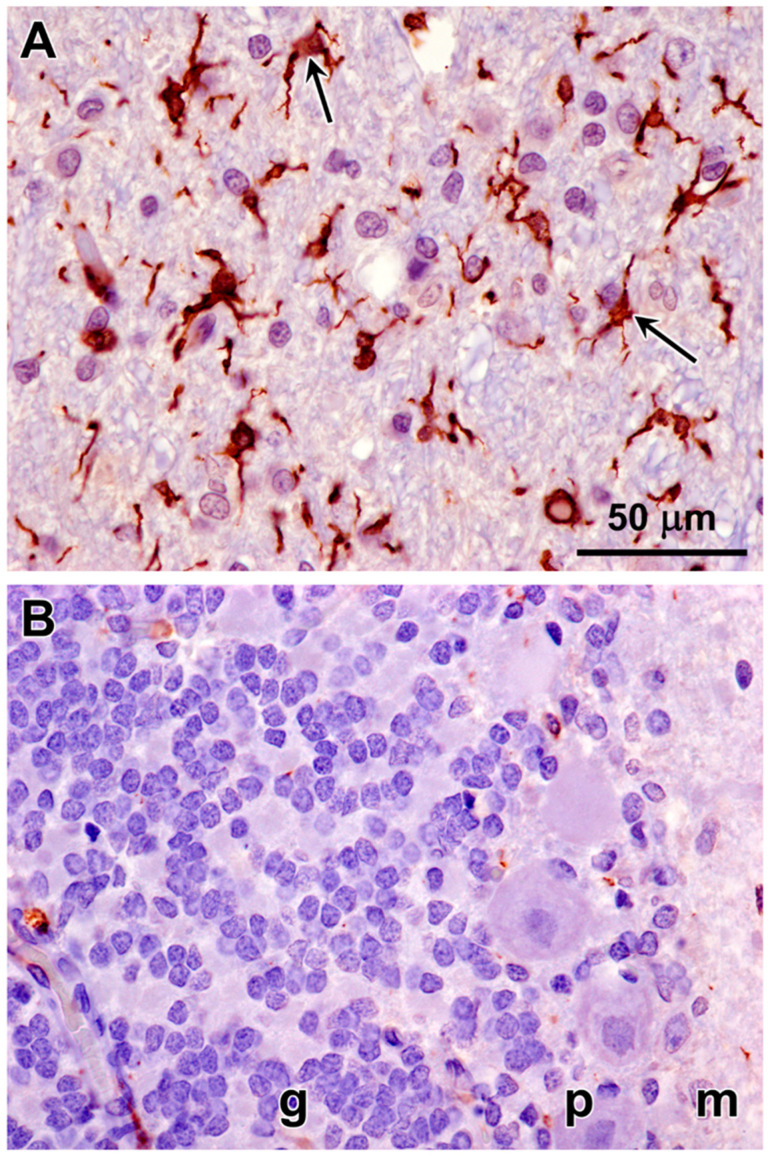
Immunohistochemical localization of the microglial activation marker Iba1 in sections of cerebral cortex (**A**) and cerebellar cortex (**B**) from the proband. Activated microglia were abundant throughout the cerebral cortex gray matter (arrows in (**A**)). Very little Iba1 immunolabeling was observed in sections of the cerebellar cortex (**B**). Layers of the cerebellar cortex: granular layer (g); Purkinje cell layer (p); molecular layer (m).

Immunolabeling of brain tissue samples of the proband indicated that the disease is characterized by neuroinflammation. Astrocyte activation, as indicated by immunolabeling for GFAP, was pronounced in both cerebral cortical gray matter and in the cerebellar cortex (Figure 21). Microglial activation, indicated by Iba1 immunolabeling, on the other hand, was pronounced in the cerebral cortex, but not in the cerebellar cortex (Figure 22).

**Figure 23:**
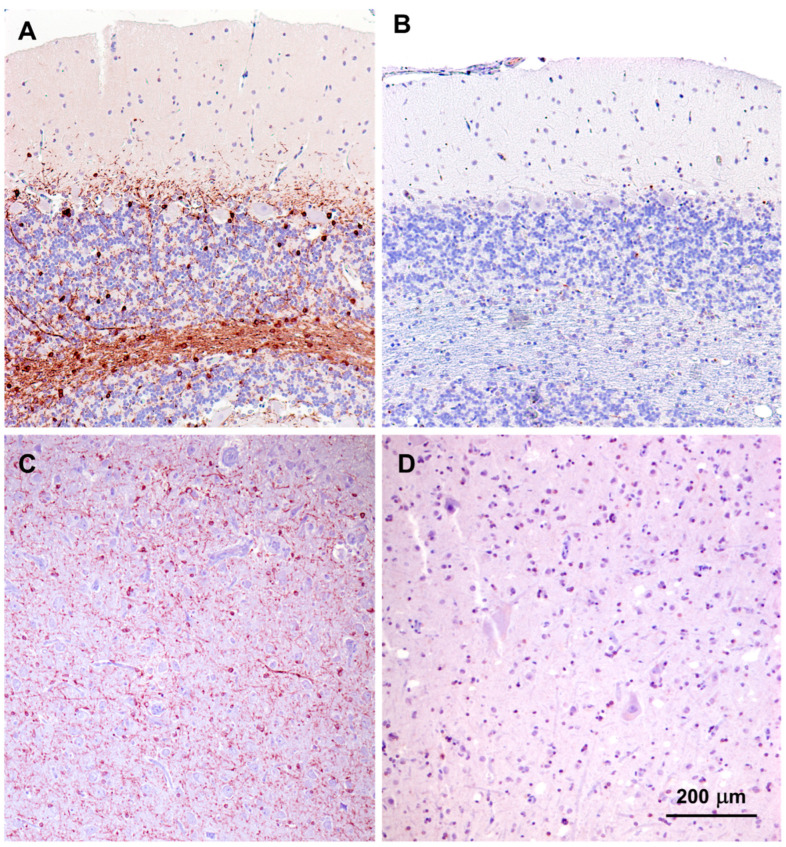
Paraffin sections of cerebellar cortex (**A**,**B**) and cerebral cortex gray matter (**C**,**D**) from an approximately 2 year old mixed breed dog with no neurological disorder (**A**,**C**) and from the proband (**B**,**C**). Sections were immunostained for localization of CNPase protein (brown color). CNPase immunolabel that was present in the tissues from the normal dog was not observed in the same tissues from the proband. Bar in (**D**) indicates magnification of all 4 micrographs.

In Dalmatians with a disorder similar to that of the proband, a frame-shift variant *CNP* that encodes 2′,3′-cyclic-nucleotide 3′-phosphodiesterase (CNPase) was accompanied by an absence of detectable CNPase protein in tissues of the affected dog [1]. Sections of cerebral cortex and cerebellar cortex from the proband, and from an unaffected dog were immunolabeled for CNPase localization. Abundant immunolabeling was observed in both tissues of the control dog but was not detectable in the tissues of the proband (Figure 23).

### 3.2. Molecular Genetic Findings

**Figure 24:**
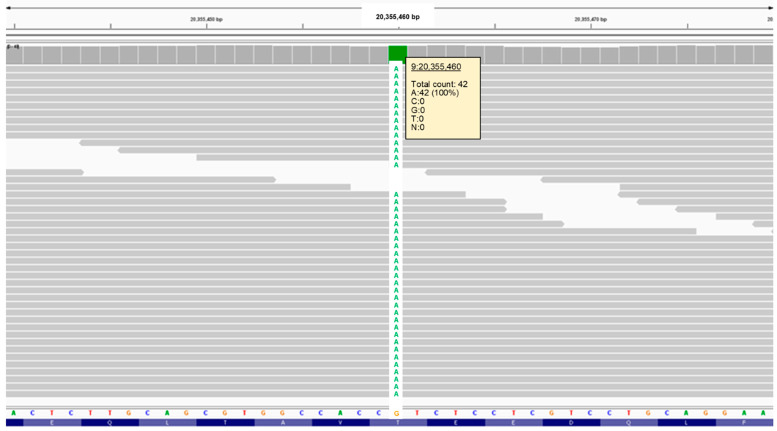
Screenshot of the proband’s whole genome sequence reads aligned to the reference sequence in the vicinity of position 20,355,460 on chromosome 9, as viewed with the Integrative Genomics Viewer. The variant A is highlighted in green.

To identify the cause of the disease, we used DNA from the proband to generate a 32.2-fold average-coverage whole-genome sequence. This sequence contained 26,761 variants relative to the canine reference sequence that were predicted to alter the primary structure of the encoded gene products. Of these, 9064 variants were in the homozygous state. When the proband’s homozygous variants were sorted according to the allele frequency among all 4035 canine whole genome sequences included in the analysis, the lowest allele frequency (0.000376 or 3 of 7986 called alleles) was a G-to-A transition at position 20,355,460 on chromosome 9, which was predicted to produce a Thr42Met missense mutation in CNPase. The validity of this variant call was confirmed by an Integrative-Genomics-Viewer-assisted inspection of aligned reads from the proband’s whole genome sequence to the Tasha reference sequence from position 20,355,440 to 20,355,479 on chromosome 9 (Figure 24) [5,6]. A previous report attributes a canine adult-onset, slowly progressive neurologic disease to a homozygous *CNP* single base deletion frame-shift variant [1]. A literature review failed to identify a similar disease phenotype in association with any of the other thirty-four genes that harbored variants with allele frequencies of 0.0003 or less. A blastp query [7] indicated that a threonine residue at an equivalent position was conserved in 100 mammalian *CNP* orthologs.

**Figure 25:**
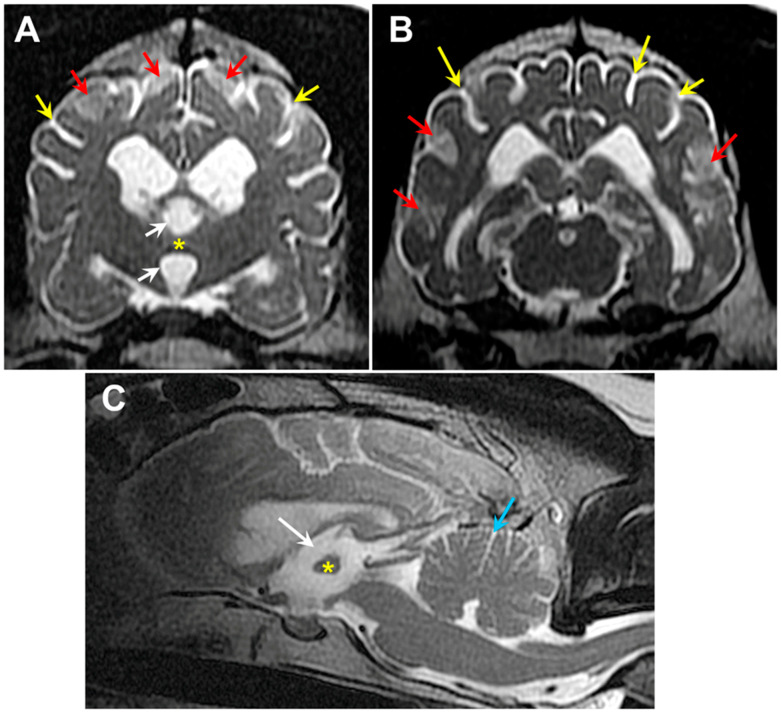
T2-weighted MR images of the brain from Dog 2: transverse views at the level of the interthalamic adhesion (**A**) and at more caudal location (**B**), and a sagittal view (**C**). The dog exhibited cerebral parenchymal atrophy characterized by an abnormally small interthalamic adhesion (yellow asterisks), an enlarged third ventricle (white arrows), and widened cerebral cortical sulci (yellow arrows). Increased CSF volume between the cerebellar folia (blue arrow) was indicative of cerebellar atrophy. The dog also exhibited multiple areas of increased signal intensity within the cerebral cortex parenchyma (red arrows).

Three hundred and four of our archived DNA samples from Weimaraners were genotyped for the 9:20,355,460 G/A polymorphism in *CNP*. In addition to the proband, one other sample tested homozygous for the variant 9:20,355,460 A allele. The homozygous sample was from a spayed female that exhibited bilateral hindlimb weakness and fecal incontinence. The sample was submitted to us to evaluate a differential diagnosis of degenerative myelopathy [8], which was ruled out when the sample tested homozygous for the reference G allele at 31:26,532,306, the site of the G-to-A transition in *SOD1* associated with degenerative myelopathy in many breeds [9,10]. When 8 years old, this Weimaraner presented for a 5-week history of pelvic limb weakness, which started after stumbling down the stairs and a one-year history of progressive fecal incontinence (dropping stools 2–3 times a week). The initial examination identified an ambulatory paraparesis and generalized whole body tremors noted at rest and during movement (a pre-existing condition present since puppyhood). T2 magnetic resonance images of the neuraxis demonstrated significant generalized brain atrophy and areas of increased signal intensity in the cerebral cortex parenchyma similar to those observed in the proband (Figure 25). Cerebrospinal fluid analysis and cytology indicated a marginal increase in protein concentration (46.0 mg/dL, upper limit RR 45 mg/dL) and normal nucleated cellularity and cell types. Genetic testing for variants previously associated with hypomyelination or spinal dysraphism demonstrated no mutations [11,12]. The patient was initially started on azathioprine (25 mg PO SID) as an empirical immunomodulatory therapy for suspected immune mediated inflammatory myopathy/polyneuropathy. Azathioprine was then discontinued due to increased hepatocellular enzyme values and cyclosporine (100 mg PO SID for 30 days), a tapering dose of prednisone (15 mg PO SID for 14 days to start), and Cerenia (30 mg PO SID PRN) therapy was initiated. Ten months later, the patient was again evaluated for increased symmetrical muscle atrophy, worsening pelvic limb paresis and generalized whole body tremors at rest and during movement or weight bearing. Because of progression in her clinical signs, the patient was euthanized, but no necropsy was performed.

Of the 304 archived Weimaraner DNA samples, nine were G/A heterozygotes at 9:20,355,460. One of these samples was from an asymptomatic 3-year-old dog. The other eight were from dogs that exhibited slowly progressive hindleg weakness and other signs of neurodegenerative disease. Their ages ranged from 8.8 years to 13.0 years (mean, 10.7 years). The samples from these eight dogs were submitted to us for degenerative myelopathy *SOD1* variant testing. All eight of these samples were found to be homozygous for the *SOD1* reference allele. The remaining 293 archived Weimaraner samples were homozygous for the *CNP* reference allele, 9:20,355,460 G. Thus, the frequency of the variant 9:20,355,460 A allele in the archived collection of Weimaraner samples was 0.021. All but 31 of the 293 Weimaraners homozygous for the *CNP* reference allele were submitted to us as degenerative myelopathy suspects because they exhibited adult-onset hindlimb weakness.

## 4. Discussion

For diagnostic purposes, we received clinical records, postmortem tissues, and blood as a source of DNA from a Weimaraner (the proband) with an adult-onset, slowly progressive neurodegenerative disease. Examination of the postmortem tissues by fluorescence-, light-, and electron-microscopy, and immunohistochemistry suggested that the dog had an LSD similar to neuronal ceroid lipofuscinosis (NCL) [13,14,15,16,17,18,19,20]; however, marked abnormalities in myelin structure distinguished the disease from most previously recognized LSDs. Examination of the proband’s whole genome sequence identified a candidate for causality: a rare homozygous missense mutation in *CNP*, the gene that encodes CNPase. This variant was recognized as a plausible causal candidate because a different homozygous *CNP* variant was known to cause a similar neurological disease of Dalmatian dogs that was also characterized by autofluorescent storage body accumulation and myelin abnormalities [1]. The absence of immunohistochemical staining of CNPase antigen in brain sections from the proband strongly supported the homozygous *CNP* missense mutation as the cause of the neurodegenerative disease.

Previous reports have described murine, human, and canine *CNP* deficiency diseases [1,21,22]. The *CNP* deficiency disease in transgenic mice was first described in 2003. *Cnp* nullizygous mice appeared normal through their first five months of life. After that, the mice developed progressive neurologic signs including ataxia, gait abnormalities, weight loss, hind-limb paralysis, convulsions, kyphosis and reduced life span. Among the post-mortem lesions were generalized brain atrophy with marked white-matter loss, axonal spheroids containing multivesicular storage bodies, myelin abnormalities including enlarged myelin inner tongues and disorganized paranodal structures, and both an astrogliosis and a microgliosis [22]. Subsequent examinations of the *Cnp* nullizygous mice found that structural myelin abnormalities preceded the overt clinical signs. Specifically, paranodal disorganization was detected at three months of age [23] and in the small-diameter fibers axonal degeneration, spheroid formation, redundant myelin loops, and swollen inner tongues (including some containing autophagic vacuoles) can be detected shortly after birth [24].

A 2020 report described an infantile-onset, progressive neurodegenerative disease in a male child with a homozygous *CNP* missense mutation identified by exome sequencing [21]. This patient appeared to have developed normally until 16 months of age, when he became abnormally irritable. After that, he exhibited delayed or regressive development. He developed microcephaly, episodic body stiffness and dystonia, scoliosis, progressive hypotonia, and died from aspiration pneumonia at five years of age. A brain MRI showed markedly reduced white matter volume. The patient’s apparently normal parents and a female sibling were heterozygous for the *CNP* missense mutation. The patient’s two earlier-born male siblings had similar clinical histories and died at seven and eight years of age. The genotypes of the latter siblings were not reported [21].

The putative causal mutation in the affected dogs predicts a substitution of methionine for threonine at position 42 in the CNPase isoform 2 (position 22 in isoform 1). Based on the immunohistochemical findings, this single amino acid substitution appears to have resulted in lack of detectable CNPase protein in brain tissues from the proband. The similar human neurological disorder that results from a Ser82Leu substitution resulted in an almost complete absence of CNPase protein in cultured cells from an affected patient relative to a healthy control subject [21]. Together, these findings suggest that both Thr42 and Ser82 are essential for maintaining the stability of the protein in both species (Supplementary File S2). In the human cells, inhibition of proteosome function did not rescue the lack of CNPase protein, indicating that the deficiency of the mutant protein was not due to accelerated degradation by proteosomes. Further research will be necessary to elucidate the mechanisms by which these amino acid substitutions destabilize the protein.

Mice that are heterozygous for a *Cnp* knockout allele appear to be phenotypically normal when younger, but by 19 months of age, these heterozygotes accumulated axonal spheroids and developed a more intense microgliosis and astrogliosis in comparison to age-matched wildtype mice [25]. In addition, these aged heterozygotes exhibited abnormal behavior that may be reflective of depression and catatonia [25]. These findings suggest that individuals of other species that are heterozygous for deleterious *CNP* variants may be at risk for developing milder later-onset neurological signs. In the current study, our archived DNA samples from Weimaraners included eight from dogs that were at least 8 years old and heterozygous for the likely causal *CNP* missense mutation. All eight exhibited hindleg weakness that was not associated with a variant in *SOD1* that underlies late-onset hindlimb weakness in many breeds [9,26,27]. It is possible that this later-onset sign is related to the *CNP* variant. It does not appear that the parents or other older relatives of the children with the *CNP*-related disorder were evaluated for subtle neurological abnormalities that might be expected based on the mouse data. Further research will be necessary to determine whether human subjects heterozygous for deleterious *CNP* variants develop late-onset neurological signs.

*CNP* encodes at least two forms of CNPase protein that have apparently unrelated functions: enzymatic catalysis of nucleoside 2′,3′-cyclic monophosphates hydrolysis to nucleoside 2′-monophosphates [28,29,30,31] and a structural role in myelin where CNPase accounts for approximately 5% of the myelin-associated protein in the central nervous system and 0.3% of the protein in the peripheral nervous system [28,29,32,33,34]. Alternative first exons and alternative initiation start sites express transcripts that encode two major CNPase isoforms, sometimes referred to as CNPase1 and CNPase2, with identical amino acid sequences except that CNPase2 has an additional 20-amino-acid extension at the N-terminal end [35,36]. When unphosphorylated, the N-terminal extension functions as a mitochondrial targeting signal [37]. Although it has been localized to the mitochondrial inner and outer membranes, the precise roles of CNPase in the mitochondria remain to be elucidated. However, studies indicate that is involved in regulating the functioning of the mitochondrial transition pore [29,38]. Abnormal regulation of the mitochondrial transition pore can result in cell damage or death [39,40,41,42]. Thus, deficiency in CNP protein would be predicted to alter both myelin structure and mitochondrial function. Clearly, the structure of the myelin sheaths surrounding axons is profoundly abnormal in both Dalmatians and Weimaraners that lack CNP as a result of different mutations, indicating that CNP protein is necessary for maintaining the normal organization of myelin sheaths. The mechanism by which it does so remains to be elucidated.

Based on ultrastructural analyses, it appears that some of the abnormal myelin is taken up into neurons and accumulates in storage bodies. Mitochondrial function was not assessed in either of the canine disorders or in the human subjects with a CNP deficiency. However, the disease-related accumulation of storage bodies of both the affected Dalmatians and Weimaraner occurs within the mitochondria-rich regions of the cardiac muscles, suggesting that these inclusions may be derived from damaged mitochondria. Storage bodies in both cardiac muscle and nervous tissue of CNP-deficient dogs of both breeds contain subunit c protein from the mitochondrial inner membrane, consistent with a mitochondrial origin of at least some of the contents of the disease-related storage bodies. Abnormal or damaged mitochondria are likely incorporated into disease-related storage bodies via autophagocytosis.

The accumulation of subunit c-containing autofluorescent storage bodies as a result of CNPase deficiency resembles that which occurs in many of the NCL disorders [13,43,44]. However, in the NCLs, the storage body accumulation is usually linked to a mutation that results in a direct impairment of lysosomal function. The CNP protein has not been shown to directly influence lysosomal function, so the storage body accumulation that occurs as a result of CNP deficiency is likely to be secondary to formation of myelin- and mitochondrial-derived substrates that become incorporated into phagolysosomes and cannot be degraded efficiently. The accumulation of these storage bodies, in addition to myelin abnormalities and mitochondrial dysfunction, may contribute to the development the neurological signs associated with CNP deficiency. The slow progression of disease signs may be explained by a gradual accumulation of storage material in neurons over time and the effects of this accumulation on cell function.

The finding that the frequency of the mutant allele among the population of Weimaraners represented in our DNA archive was almost 2% suggests that there are likely to be additional affected dogs in the pet population. Now that the causal mutation has been identified, breeders can have their dogs genotyped for the variant allele and thereby avoid producing affected dogs in the future. This type of genetic screening for disease-causing mutations has greatly reduced the incidence of numerous genetic diseases in the canine pet population.

## Data Availability

DNA sequence data for the proband have been archived and deposited in the NCBI Sequence Read Archive as BioSample SAMN24256744.

## Supplementary Materials

The following supporting information can be downloaded at: https://www.mdpi.com/article/10.3390/genes15020246/s1, Supplementary File S1 consists of a list of the NCBI Sequence Read Archive BioSample identifiers for the whole genome sequences compared in this study. Supplementary File S2 shows the alignment of the human and canine CNPase2 amino acid sequences and the locations of the amino acid substitutions associated with the neurological disorders.
