# Supplementary material for: Homozygous CNP Mutation and Neurodegeneration in Weimaraners: Myelin Abnormalities and Accumulation of Lipofuscin-like Inclusions
Source: Genes (Basel). 2024 Feb 15;15(2):246. doi: 10.3390/genes15020246 (PMC10888007; doi:10.3390/genes15020246)
Supplement: Supplementary file 1 [file genes-15-00246-s001.zip › Supplementary File S1-CNP-WEIM.pdf]

Supplementary File S1 _: SRA BioSample IDs for the 4,062 sample cohort, sorted in ascen

BioSample ID
SAMN24256744
SAMN03580379
SAMN03580380
SAMN03580381
SAMN04196845
SAMN03323670
SAMN04196846
SAMN03323672
SAMN04196847
SAMN04196848
SAMN03580382
SAMN03145702
SAMN03145703
SAMN03580383
SAMN03580384
SAMN03580385
SAMN03580386
SAMN03580387
SAMN03580388
SAMN03580389
SAMN03580390
SAMN03580391
SAMN03580392
SAMN03580393
SAMN10940760
SAMN10940761
SAMN10940762
SAMN03580394
SAMN03580395
SAMN03580396
SAMN03580397
SAMN03580398
SAMN03580399
SAMN03323668
SAMN03580400
SAMN03580401
SAMN03580402
SAMN03580403
SAMN03580404
SAMN03580405
SAMN03580406

SAMN03580407
SAMN03580408
SAMN03145704
SAMN10940763
SAMN10940764
SAMN03580409
SAMN10940765
SAMN03580410
SAMN03580411
SAMN03580412
SAMN03075611
SAMN03580413
SAMN03323676
SAMN04196849
SAMN04196850
SAMN03323671
SAMN04196851
SAMN04196852
SAMN03323673
SAMN04196853
SAMN03323674
SAMN04196854
SAMN04196855
SAMN04196856
SAMN04196857
SAMN04196858
SAMN04196859
SAMN04196860
SAMN03323675
SAMN04196861
SAMN04196862
SAMN03323669
SAMN03323677
SAMN04196863
SAMN04196864
SAMN04196865
SAMN06159667
SAMN06159668
SAMN06159669
SAMN06159670
SAMN06159671
SAMN06159672
SAMN06159673
SAMN05770194

SAMN06159674
SAMN06159675
SAMN06159676
SAMN06159677
SAMN06159678
SAMN06159679
SAMN06159680
SAMN06159681
SAMN06159682
SAMN06159683
SAMN06159684
SAMN06159685
SAMN06159686
SAMN06159687
SAMN06159688
SAMN06159689
SAMN10940769
SAMN10940770
SAMN10940771
SAMN10940772
SAMN10940773
SAMN10940774
SAMN10940775
SAMN10940776
SAMN10940777
SAMN10940778
SAMN10940779
SAMN10940780
SAMN10940781
SAMN10940782
SAMN10940783
SAMN10940784
SAMN10940785
SAMN10940786
SAMN10940787
SAMN10940788
SAMN10940789
SAMN10940790
SAMN10940791
SAMN10940792
SAMN10940793
SAMN10940794
SAMN10940795
SAMN10940796

SAMN10940797
SAMN10940798
SAMN10940799
SAMN10940800
SAMN10940801
SAMN10940802
SAMN10940803
SAMN10940804
SAMN10940805
SAMN10940806
SAMN10940807
SAMN10940808
SAMN10940809
SAMN10940810
SAMN10940811
SAMN10940812
SAMN10940813
SAMN10940814
SAMN10940815
SAMN10940816
SAMN10940817
SAMN10940818
SAMN10940819
SAMN10940820
SAMN10940821
SAMN10940822
SAMN10940823
SAMN10940824
SAMN10940825
SAMN10940826
SAMN10940827
SAMN12700520
SAMN12700521
SAMN12700522
SAMN12700523
SAMN12700524
SAMN12700525
SAMN12700526
SAMN12700527
SAMN12700528
SAMN12700529
SAMN12700530
SAMN12700531
SAMN12700532

SAMN12700533
SAMN12700534
SAMN12700535
SAMN12700536
SAMN12700537
SAMN12700538
SAMN12700539
SAMN12700540
SAMN12700541
SAMN12700542
SAMN12700543
SAMN12700544
SAMN12700545
SAMN12700546
SAMN12700547
SAMN12700548
SAMN12700549
SAMN12700550
SAMN12700551
SAMN12700552
SAMN12700553
SAMN12700554
SAMN12700555
SAMN13655888
SAMN13655889
SAMN13655890
SAMN13655891
SAMN13655892
SAMN13655893
SAMN13655894
SAMN13655895
SAMN13655896
SAMN13655897
SAMN13655898
SAMN13655899
SAMN13655900
SAMN13655901
SAMN13655902
SAMN13655903
SAMN13655904
SAMN13655905
SAMN13655906
SAMN13655907
SAMN13655908

SAMN13655909
SAMN13655910
SAMN13655911
SAMN13655912
SAMN13655913
SAMN13655914
SAMN13655915
SAMN13655916
SAMN13655917
SAMN13655918
SAMN13655919
SAMN13655920
SAMN13655921
SAMN13655922
SAMN13655923
SAMN24256719
SAMN24256720
SAMN24256721
SAMN24256722
SAMN24256723
SAMN24256724
SAMN24256725
SAMN24256726
SAMN24256727
SAMN24256728
SAMN24256729
SAMN24256730
SAMN24256731
SAMN24256732
SAMN24256733
SAMN24256734
SAMN24256735
SAMN24256736
SAMN24256737
SAMN24256738
SAMN24256739
SAMN24256740
SAMN24256741
SAMN24256742
SAMN24256743
SAMN24256745
SAMD00009664
SAMEA103135918
SAMEA103935360

SAMEA103949042
SAMEA104016307
SAMEA104032048
SAMEA104091550
SAMEA104091551
SAMEA104091552
SAMEA104091553
SAMEA104091554
SAMEA104091555
SAMEA104091556
SAMEA104091557
SAMEA104091558
SAMEA104091559
SAMEA104091560
SAMEA104091561
SAMEA104091562
SAMEA104091563
SAMEA104091564
SAMEA104091565
SAMEA104091566
SAMEA104091567
SAMEA104091568
SAMEA104091569
SAMEA104091570
SAMEA104091571
SAMEA104091572
SAMEA104091573
SAMEA104105252
SAMEA104125075
SAMEA104125112
SAMEA104125113
SAMEA104125115
SAMEA104125116
SAMEA104125117
SAMEA104125118
SAMEA104125119
SAMEA104125120
SAMEA104125121
SAMEA104140396
SAMEA104140397
SAMEA104140398
SAMEA104190273
SAMEA104211056
SAMEA104211057

SAMEA104211058
SAMEA104345830
SAMEA104379879
SAMEA104379880
SAMEA104379881
SAMEA104379882
SAMEA104379883
SAMEA104379884
SAMEA104379885
SAMEA104388841
SAMEA104388842
SAMEA104388843
SAMEA104388844
SAMEA104388845
SAMEA104388846
SAMEA104388847
SAMEA104388848
SAMEA104388849
SAMEA104388850
SAMEA104388851
SAMEA104389201
SAMEA104389202
SAMEA104389203
SAMEA104389204
SAMEA104389205
SAMEA104389206
SAMEA104389207
SAMEA104389208
SAMEA104389209
SAMEA104389210
SAMEA104389211
SAMEA104389212
SAMEA104389213
SAMEA104389237
SAMEA104389238
SAMEA104389239
SAMEA104389240
SAMEA104389241
SAMEA104389242
SAMEA104389243
SAMEA104427625
SAMEA104500411
SAMEA104500412
SAMEA104500413

SAMEA104656180
SAMEA104656181
SAMEA104656182
SAMEA104656183
SAMEA104658568
SAMEA104658569
SAMEA104658570
SAMEA1066474
SAMEA1066475
SAMEA1066476
SAMEA1521940
SAMEA1521941
SAMEA1561463
SAMEA1681455
SAMEA1681463
SAMEA1681464
SAMEA1681469
SAMEA1681472
SAMEA2177732
SAMEA2358413
SAMEA2358414
SAMEA2358415
SAMEA2358416
SAMEA2376414
SAMEA2417015
SAMEA2417016
SAMEA2446055
SAMEA2446720
SAMEA3121328
SAMEA3121337
SAMEA3121338
SAMEA3164479
SAMEA3402917
SAMEA3449656
SAMEA3449657
SAMEA3539249
SAMEA3723573
SAMEA3724570
SAMEA3724571
SAMEA3905753
SAMEA3906811
SAMEA3906812
SAMEA3906815
SAMEA3906823

SAMEA3906850
SAMEA3906876
SAMEA3906879
SAMEA3906880
SAMEA3906883
SAMEA3906885
SAMEA3906887
SAMEA3906889
SAMEA3906893
SAMEA3906895
SAMEA3906897
SAMEA3906903
SAMEA3906904
SAMEA3906905
SAMEA3906906
SAMEA3906909
SAMEA3906910
SAMEA3906911
SAMEA3906912
SAMEA3906913
SAMEA3906916
SAMEA3906918
SAMEA3906919
SAMEA3906921
SAMEA3906923
SAMEA3906926
SAMEA3906927
SAMEA3906931
SAMEA3906933
SAMEA3906946
SAMEA3906956
SAMEA3906959
SAMEA3906961
SAMEA3906965
SAMEA3906973
SAMEA3906979
SAMEA3906982
SAMEA3906986
SAMEA3906989
SAMEA3906992
SAMEA3906993
SAMEA3906994
SAMEA3907000
SAMEA3907006

SAMEA3907009
SAMEA3907010
SAMEA3907011
SAMEA3907012
SAMEA3907014
SAMEA3907016
SAMEA3907017
SAMEA3907018
SAMEA3907019
SAMEA3907020
SAMEA3907022
SAMEA3907028
SAMEA3907030
SAMEA3907032
SAMEA3907035
SAMEA3907038
SAMEA3907040
SAMEA3907043
SAMEA3907044
SAMEA3907045
SAMEA3907046
SAMEA3907048
SAMEA3907052
SAMEA3907058
SAMEA3907061
SAMEA3907064
SAMEA3907067
SAMEA3907069
SAMEA3907071
SAMEA3907073
SAMEA3907076
SAMEA3907083
SAMEA3907180
SAMEA3907184
SAMEA3907186
SAMEA3907187
SAMEA3907188
SAMEA3907189
SAMEA3907190
SAMEA3907191
SAMEA3907192
SAMEA3907193
SAMEA3907198
SAMEA3907199

SAMEA3907203
SAMEA3907205
SAMEA3907207
SAMEA3907208
SAMEA3907212
SAMEA3907215
SAMEA3907217
SAMEA3907221
SAMEA3907222
SAMEA3907231
SAMEA3907234
SAMEA3907237
SAMEA3907238
SAMEA3907241
SAMEA3907243
SAMEA3907260
SAMEA3907264
SAMEA3907267
SAMEA3907271
SAMEA3907276
SAMEA3907279
SAMEA3907280
SAMEA3907281
SAMEA3907282
SAMEA3907286
SAMEA3907287
SAMEA3907289
SAMEA3907293
SAMEA3907304
SAMEA3907305
SAMEA3907306
SAMEA3907362
SAMEA3907363
SAMEA3907365
SAMEA3907368
SAMEA3907369
SAMEA3907380
SAMEA3907382
SAMEA3907388
SAMEA3907389
SAMEA3907400
SAMEA3907401
SAMEA3907403
SAMEA3907407

SAMEA3907409
SAMEA3907411
SAMEA3907412
SAMEA3907413
SAMEA3907414
SAMEA3907419
SAMEA3907420
SAMEA3907421
SAMEA3907427
SAMEA3907428
SAMEA3910718
SAMEA3910719
SAMEA3910747
SAMEA3910756
SAMEA3910758
SAMEA3928139
SAMEA3928140
SAMEA3928141
SAMEA3928142
SAMEA3928143
SAMEA3928144
SAMEA3928145
SAMEA3928146
SAMEA3940300
SAMEA3994036
SAMEA3994037
SAMEA3994038
SAMEA4040708
SAMEA4040709
SAMEA4040710
SAMEA4040711
SAMEA4346711
SAMEA4504820
SAMEA4504821
SAMEA4504822
SAMEA4504823
SAMEA4504824
SAMEA4504825
SAMEA4504826
SAMEA4504827
SAMEA4504828
SAMEA4504829
SAMEA4504830
SAMEA4504831

SAMEA4504832
SAMEA4504833
SAMEA4504834
SAMEA4504835
SAMEA4504837
SAMEA4504838
SAMEA4504839
SAMEA4504840
SAMEA4505489
SAMEA4505490
SAMEA4505491
SAMEA4505492
SAMEA4505493
SAMEA4505494
SAMEA4505495
SAMEA4505496
SAMEA4505497
SAMEA4505498
SAMEA4505499
SAMEA4505500
SAMEA4505501
SAMEA4506885
SAMEA4506886
SAMEA4506887
SAMEA4506888
SAMEA4506889
SAMEA4506890
SAMEA4506891
SAMEA4506892
SAMEA4506893
SAMEA4506894
SAMEA4506895
SAMEA4506896
SAMEA4506897
SAMEA4506898
SAMEA4506899
SAMEA4506900
SAMEA4506901
SAMEA4506902
SAMEA4509487
SAMEA4509488
SAMEA4509489
SAMEA4509490
SAMEA4509491

SAMEA4509492
SAMEA4672966
SAMEA4672967
SAMEA4672968
SAMEA4672969
SAMEA4823343
SAMEA4823353
SAMEA4823354
SAMEA4823355
SAMEA4823356
SAMEA4823357
SAMEA4823358
SAMEA4823360
SAMEA4848665
SAMEA4848666
SAMEA4848667
SAMEA4848669
SAMEA4848670
SAMEA4848671
SAMEA4848672
SAMEA4848673
SAMEA4848674
SAMEA4848675
SAMEA4848676
SAMEA4848677
SAMEA4848678
SAMEA4848679
SAMEA4848680
SAMEA4848681
SAMEA4848682
SAMEA4848683
SAMEA4848690
SAMEA4848691
SAMEA4848692
SAMEA4848693
SAMEA4848694
SAMEA4848695
SAMEA4848696
SAMEA4848697
SAMEA4848698
SAMEA4848700
SAMEA4848701
SAMEA4848702
SAMEA4848703

SAMEA4848704
SAMEA4848705
SAMEA4848706
SAMEA4848707
SAMEA4848708
SAMEA4848709
SAMEA4848710
SAMEA4848711
SAMEA4848712
SAMEA4848713
SAMEA4848714
SAMEA4867919
SAMEA4867920
SAMEA4867921
SAMEA4867922
SAMEA4867923
SAMEA4867924
SAMEA4867925
SAMEA4867926
SAMEA4867927
SAMEA4867928
SAMEA4867929
SAMEA4867930
SAMEA4867931
SAMEA4867932
SAMEA4867933
SAMEA4867934
SAMEA4867935
SAMEA4867936
SAMEA4873254
SAMEA4949388
SAMEA4949389
SAMEA4949390
SAMEA4966024
SAMEA4966025
SAMEA5059499
SAMEA5059500
SAMEA5059501
SAMEA5059502
SAMEA5059503
SAMEA5059504
SAMEA5059505
SAMEA5059506
SAMEA5059507

SAMEA5059508
SAMEA5059509
SAMEA5059510
SAMEA5059511
SAMEA5059514
SAMEA5059515
SAMEA5059516
SAMEA5059517
SAMEA5059518
SAMEA5059520
SAMEA5059521
SAMEA5059522
SAMEA5059523
SAMEA5205215
SAMEA5205216
SAMEA5205217
SAMEA5205218
SAMEA5205219
SAMEA5205220
SAMEA5205221
SAMEA5205222
SAMEA5205223
SAMEA5205224
SAMEA5205225
SAMEA5205226
SAMEA5205227
SAMEA5205228
SAMEA5205229
SAMEA5560215
SAMEA5560216
SAMEA5560217
SAMEA5560218
SAMEA5577245
SAMEA5577246
SAMEA5657392
SAMEA5657394
SAMEA5657395
SAMEA5657396
SAMEA5657397
SAMEA5657398
SAMEA5657399
SAMEA5657400
SAMEA5818551
SAMEA5818552

SAMEA676101
SAMEA676105
SAMEA676114
SAMEA676121
SAMEA676122
SAMEA72802168
SAMEA72802918
SAMN01737209
SAMN01893932
SAMN01908107
SAMN01974486
SAMN01974487
SAMN01974488
SAMN01974489
SAMN01974490
SAMN01974491
SAMN01974492
SAMN01974493
SAMN01974494
SAMN01974495
SAMN02144216
SAMN02144219
SAMN02194722
SAMN02194723
SAMN02485564
SAMN02485565
SAMN02485566
SAMN02485567
SAMN02485568
SAMN02485569
SAMN02485570
SAMN02485571
SAMN02485572
SAMN02485573
SAMN02485574
SAMN02485575
SAMN02485576
SAMN02485577
SAMN02485578
SAMN02485579
SAMN02485580
SAMN02485581
SAMN02485582
SAMN02485583

SAMN02485584
SAMN02485585
SAMN02485586
SAMN02485587
SAMN02485588
SAMN02485589
SAMN02485590
SAMN02485591
SAMN02485592
SAMN02485593
SAMN02485594
SAMN02485595
SAMN02485596
SAMN02485597
SAMN02485598
SAMN02485599
SAMN02485600
SAMN02485601
SAMN02485602
SAMN02485603
SAMN02485604
SAMN02485605
SAMN02485606
SAMN02485607
SAMN02485608
SAMN02485609
SAMN02485610
SAMN02485611
SAMN02485612
SAMN02485613
SAMN02485614
SAMN02570458
SAMN02585152
SAMN02585153
SAMN02585154
SAMN02585155
SAMN02585156
SAMN02585157
SAMN02585158
SAMN02585159
SAMN02585160
SAMN02585161
SAMN02585162
SAMN02585163

SAMN02585164
SAMN02585165
SAMN02585166
SAMN02585167
SAMN02585168
SAMN02585169
SAMN02585170
SAMN02585171
SAMN02585172
SAMN02585173
SAMN02585174
SAMN02585175
SAMN02585176
SAMN02585177
SAMN02585178
SAMN02585179
SAMN02585180
SAMN02585181
SAMN02585182
SAMN02585183
SAMN02585184
SAMN02585185
SAMN02585186
SAMN02585187
SAMN02585188
SAMN02585189
SAMN02585190
SAMN02585191
SAMN02585192
SAMN02585193
SAMN02585194
SAMN02585195
SAMN02585196
SAMN02585197
SAMN02585198
SAMN02585199
SAMN02585200
SAMN02585201
SAMN02585202
SAMN02585203
SAMN02585204
SAMN02585205
SAMN02585206
SAMN02585207

SAMN02585208
SAMN02585209
SAMN02585210
SAMN02921304
SAMN02921305
SAMN02921306
SAMN02921307
SAMN02921308
SAMN02921309
SAMN03067872
SAMN03067873
SAMN03067874
SAMN03067875
SAMN03067876
SAMN03067877
SAMN03067878
SAMN03067879
SAMN03067880
SAMN03067881
SAMN03067882
SAMN03067883
SAMN03067884
SAMN03067890
SAMN03067891
SAMN03067893
SAMN03067894
SAMN03067895
SAMN03067896
SAMN03068228
SAMN03068229
SAMN03068230
SAMN03068231
SAMN03168352
SAMN03168353
SAMN03168354
SAMN03168355
SAMN03168356
SAMN03168357
SAMN03168358
SAMN03168359
SAMN03168360
SAMN03168361
SAMN03168363
SAMN03168364

SAMN03168365
SAMN03168366
SAMN03168367
SAMN03168368
SAMN03168369
SAMN03168370
SAMN03168371
SAMN03168372
SAMN03168373
SAMN03168374
SAMN03168375
SAMN03168376
SAMN03168377
SAMN03168378
SAMN03168379
SAMN03168380
SAMN03168381
SAMN03168382
SAMN03168383
SAMN03168384
SAMN03168385
SAMN03168386
SAMN03168387
SAMN03168388
SAMN03168389
SAMN03168390
SAMN03168391
SAMN03168392
SAMN03275921
SAMN03275922
SAMN03275923
SAMN03275924
SAMN03366708
SAMN03801643
SAMN03801644
SAMN03801645
SAMN03801646
SAMN03801647
SAMN03801648
SAMN03801649
SAMN03801650
SAMN03801651
SAMN03801652
SAMN03801653

SAMN03801654
SAMN03801655
SAMN03801656
SAMN03801657
SAMN03801658
SAMN03801659
SAMN03801660
SAMN03801661
SAMN03801662
SAMN03801663
SAMN03801664
SAMN03801665
SAMN03801666
SAMN03801667
SAMN03801668
SAMN03801669
SAMN03801670
SAMN03801671
SAMN03801672
SAMN03801673
SAMN03801674
SAMN03801675
SAMN03801676
SAMN03801677
SAMN03801678
SAMN03801679
SAMN03801680
SAMN03801681
SAMN03801682
SAMN03801683
SAMN03801684
SAMN03801685
SAMN03801686
SAMN03801687
SAMN03801688
SAMN03801689
SAMN03801690
SAMN03801691
SAMN03801692
SAMN03801693
SAMN03801694
SAMN04195504
SAMN04195505
SAMN04195506

SAMN04195507
SAMN04195508
SAMN04195509
SAMN04195510
SAMN04440505
SAMN04440506
SAMN04851101
SAMN04851103
SAMN04851104
SAMN04852950
SAMN04852951
SAMN04852952
SAMN04852953
SAMN04852954
SAMN04852955
SAMN04852956
SAMN04852957
SAMN04852958
SAMN04852959
SAMN04852960
SAMN04852961
SAMN04884534
SAMN04884535
SAMN04908307
SAMN04908308
SAMN04908309
SAMN04908310
SAMN04908311
SAMN05185373
SAMN05185377
SAMN05185379
SAMN05185380
SAMN05185382
SAMN05185384
SAMN05185385
SAMN05185393
SAMN05185394
SAMN05185396
SAMN05185397
SAMN05185399
SAMN05185400
SAMN05185401
SAMN05185404
SAMN05356426

SAMN05356427
SAMN05468142
SAMN05468143
SAMN05831101
SAMN05831102
SAMN05831103
SAMN05831104
SAMN06128270
SAMN06128271
SAMN06128272
SAMN06128273
SAMN06161404
SAMN06167373
SAMN06167374
SAMN06167375
SAMN06167376
SAMN06167377
SAMN06167378
SAMN06167379
SAMN06167380
SAMN06167381
SAMN06167382
SAMN06167383
SAMN06167384
SAMN06167385
SAMN06167386
SAMN06167387
SAMN06167388
SAMN06167389
SAMN06167390
SAMN06167391
SAMN06167392
SAMN06167393
SAMN06167394
SAMN06214558
SAMN06244664
SAMN06244665
SAMN06244666
SAMN06244667
SAMN06244668
SAMN06608432
SAMN06608434
SAMN06608444
SAMN06647475

SAMN06853971
SAMN06853972
SAMN06853973
SAMN06853974
SAMN06853975
SAMN07205120
SAMN07205121
SAMN07205122
SAMN07205123
SAMN07205124
SAMN07205125
SAMN07313075
SAMN07360925
SAMN07360926
SAMN07376261
SAMN07376262
SAMN07376263
SAMN07376264
SAMN07376265
SAMN07376266
SAMN07376267
SAMN07376268
SAMN07376269
SAMN07376270
SAMN07376271
SAMN07376272
SAMN07376273
SAMN07419816
SAMN08032746
SAMN08035651
SAMN08035652
SAMN08035653
SAMN08035654
SAMN08035655
SAMN08035656
SAMN08035657
SAMN08035658
SAMN08035659
SAMN08035660
SAMN08132858
SAMN08177877
SAMN08872810
SAMN08872813
SAMN08872843

SAMN08872844
SAMN08872845
SAMN08872846
SAMN08872847
SAMN08872848
SAMN08872849
SAMN08872850
SAMN08872851
SAMN08872852
SAMN08872853
SAMN08872854
SAMN08872855
SAMN08872856
SAMN08872857
SAMN08872858
SAMN08872859
SAMN08872866
SAMN08872867
SAMN08872868
SAMN08872869
SAMN08872870
SAMN08872871
SAMN08872872
SAMN08872873
SAMN08872874
SAMN08872875
SAMN08872876
SAMN08872877
SAMN08872878
SAMN08872879
SAMN08872880
SAMN08872881
SAMN08872882
SAMN08872905
SAMN08872906
SAMN08872908
SAMN08872909
SAMN08872913
SAMN08872914
SAMN08872915
SAMN08872916
SAMN08872917
SAMN08872918
SAMN08872919

SAMN08872920
SAMN08872926
SAMN08872976
SAMN08873011
SAMN08873012
SAMN08873033
SAMN08873034
SAMN08873035
SAMN08873036
SAMN08873037
SAMN08873038
SAMN08873062
SAMN08873112
SAMN08873115
SAMN08873116
SAMN08873117
SAMN08873128
SAMN08873130
SAMN08873139
SAMN08873141
SAMN08873142
SAMN08873143
SAMN08873144
SAMN08873145
SAMN08873146
SAMN08873147
SAMN08873148
SAMN08873155
SAMN08873160
SAMN08873161
SAMN08873162
SAMN08873174
SAMN08873175
SAMN08873176
SAMN08873177
SAMN08873188
SAMN08873199
SAMN08873219
SAMN08873220
SAMN08873221
SAMN08873222
SAMN08873223
SAMN08873224
SAMN08873225

SAMN08873226
SAMN08873227
SAMN08873228
SAMN08873229
SAMN08873243
SAMN08873244
SAMN08873245
SAMN08873246
SAMN08873247
SAMN08873251
SAMN08873258
SAMN08873262
SAMN08873264
SAMN08873270
SAMN08873318
SAMN08873319
SAMN08873321
SAMN08873322
SAMN08873323
SAMN08873328
SAMN08873329
SAMN08873330
SAMN08873331
SAMN08873332
SAMN08873333
SAMN08873334
SAMN08873335
SAMN08873336
SAMN08873337
SAMN08873338
SAMN08873339
SAMN08873423
SAMN08873428
SAMN08873429
SAMN08873447
SAMN08873448
SAMN08873449
SAMN08873450
SAMN08873451
SAMN08873452
SAMN08873453
SAMN08873454
SAMN08873455
SAMN08873456

SAMN08873457
SAMN08873458
SAMN08873459
SAMN08873460
SAMN08873461
SAMN08873462
SAMN08873463
SAMN08873464
SAMN08873465
SAMN08873466
SAMN08873467
SAMN08873468
SAMN08873469
SAMN08873470
SAMN08873471
SAMN08873472
SAMN08873473
SAMN08873474
SAMN08873475
SAMN08873476
SAMN08873477
SAMN08873478
SAMN08873479
SAMN08873480
SAMN08873481
SAMN08873482
SAMN08873483
SAMN08873484
SAMN08873485
SAMN08873486
SAMN08873487
SAMN08873488
SAMN08873489
SAMN08873490
SAMN08873491
SAMN08873492
SAMN08873493
SAMN08873494
SAMN08873495
SAMN08873496
SAMN08873497
SAMN08873498
SAMN08873499
SAMN08873500

SAMN08873501
SAMN08873502
SAMN08873503
SAMN08873504
SAMN08873505
SAMN08873506
SAMN08873507
SAMN08873508
SAMN08873509
SAMN08873510
SAMN08873511
SAMN08873512
SAMN08873513
SAMN08873515
SAMN08873516
SAMN08873517
SAMN08873518
SAMN08873519
SAMN09430724
SAMN09430725
SAMN09430726
SAMN09430727
SAMN09435548
SAMN09435549
SAMN09845873
SAMN09845874
SAMN09882737
SAMN09882738
SAMN09882739
SAMN10077097
SAMN10077098
SAMN10077099
SAMN10167127
SAMN10174932
SAMN10174933
SAMN10174936
SAMN10386546
SAMN10386547
SAMN10386548
SAMN10386549
SAMN10386550
SAMN10386551
SAMN10386552
SAMN10386553

SAMN10386554
SAMN10386555
SAMN10386556
SAMN10386557
SAMN10386558
SAMN10386559
SAMN10584881
SAMN10740936
SAMN10740937
SAMN10740938
SAMN10740939
SAMN10740940
SAMN10740941
SAMN10740942
SAMN10740943
SAMN10740944
SAMN10740945
SAMN10740946
SAMN10740947
SAMN10740948
SAMN10740949
SAMN10740950
SAMN10740951
SAMN10740952
SAMN10740953
SAMN10740954
SAMN10740955
SAMN10740956
SAMN10740957
SAMN10740958
SAMN10740959
SAMN10740960
SAMN10740961
SAMN10740962
SAMN10740963
SAMN10740964
SAMN10740965
SAMN10740966
SAMN10740967
SAMN10740968
SAMN10740969
SAMN10740970
SAMN10740971
SAMN10740972

SAMN10740973
SAMN10740974
SAMN10740975
SAMN10740976
SAMN10740977
SAMN10740978
SAMN10740979
SAMN10740980
SAMN10740981
SAMN10740982
SAMN10740983
SAMN10740984
SAMN10740985
SAMN10740986
SAMN10740987
SAMN10740988
SAMN10740989
SAMN10740990
SAMN10740991
SAMN10740992
SAMN10740993
SAMN10740994
SAMN10740995
SAMN10740996
SAMN10740997
SAMN10740998
SAMN10740999
SAMN10741000
SAMN10741001
SAMN10741002
SAMN10741003
SAMN10741004
SAMN10741005
SAMN10741006
SAMN10741010
SAMN10741011
SAMN10741012
SAMN10741013
SAMN10741014
SAMN10741015
SAMN10741016
SAMN10741017
SAMN10741018
SAMN10741019

SAMN10741020
SAMN10741021
SAMN10741022
SAMN10741023
SAMN10741024
SAMN10741025
SAMN10741026
SAMN11079481
SAMN11079482
SAMN11079484
SAMN11079485
SAMN11079487
SAMN11079488
SAMN11079490
SAMN11079491
SAMN11079493
SAMN11079494
SAMN11079496
SAMN11079499
SAMN11079500
SAMN11079502
SAMN11079503
SAMN11079505
SAMN11079506
SAMN11079508
SAMN11079509
SAMN11079511
SAMN11079512
SAMN11079514
SAMN11079515
SAMN11079517
SAMN11079518
SAMN11079520
SAMN11079521
SAMN11079523
SAMN11079524
SAMN11079526
SAMN11079527
SAMN11079529
SAMN11079530
SAMN11079532
SAMN11079533
SAMN11079535
SAMN11079536

SAMN11079538
SAMN11079539
SAMN11079541
SAMN11079542
SAMN11079544
SAMN11079545
SAMN11079547
SAMN11079548
SAMN11079550
SAMN11079551
SAMN11526750
SAMN11526776
SAMN11526802
SAMN11526829
SAMN11775869
SAMN11843684
SAMN11843695
SAMN11843698
SAMN11843701
SAMN11843702
SAMN12347403
SAMN12567870
SAMN12567871
SAMN12667790
SAMN12999906
SAMN12999907
SAMN12999908
SAMN12999909
SAMN12999910
SAMN12999911
SAMN13133746
SAMN13133747
SAMN13133748
SAMN13133749
SAMN13133750
SAMN13133751
SAMN13133752
SAMN13133753
SAMN13133754
SAMN13133755
SAMN13133756
SAMN13133757
SAMN13133758
SAMN13133759

SAMN13133760
SAMN13133761
SAMN13133762
SAMN13133763
SAMN13133764
SAMN13133765
SAMN13133766
SAMN13133767
SAMN13133768
SAMN13133769
SAMN13133770
SAMN13133771
SAMN13133772
SAMN13133773
SAMN13133774
SAMN13133775
SAMN13133776
SAMN13133777
SAMN13133778
SAMN13133779
SAMN13133780
SAMN13133781
SAMN13133782
SAMN13133783
SAMN13133784
SAMN13133785
SAMN13133786
SAMN13133787
SAMN13133788
SAMN13133789
SAMN13133790
SAMN13133791
SAMN13133792
SAMN13133793
SAMN13133794
SAMN13133795
SAMN13133796
SAMN13133797
SAMN13133798
SAMN13133799
SAMN13133800
SAMN13133801
SAMN13133802
SAMN13133803

SAMN13133804
SAMN13133805
SAMN13133806
SAMN13133807
SAMN13133808
SAMN13133809
SAMN13133810
SAMN13133811
SAMN13133812
SAMN13133813
SAMN13133814
SAMN13133815
SAMN13133816
SAMN13133817
SAMN13133818
SAMN13133819
SAMN13133820
SAMN13133821
SAMN13133822
SAMN13133823
SAMN13133824
SAMN13133825
SAMN13133826
SAMN13164793
SAMN13164794
SAMN13672482
SAMN13672483
SAMN12565076
SAMN12565083
SAMN12565067
SAMN12565068
SAMN12565084
SAMN12565082
SAMN12565075
SAMN12565069
SAMN12565070
SAMN12565071
SAMN12565077
SAMEA5533140
SAMEA5533142
SAMEA5533152
SAMEA5533154
SAMEA5533158
SAMEA6862978

SAMN14210382
SAMN14210383
SAMEA6862930
SAMEA6862931
SAMEA6862932
SAMEA6862933
SAMEA6862934
SAMEA6862935
SAMEA6862936
SAMEA6862937
SAMEA6862957
SAMEA6862979
SAMEA6862929
SAMEA6862980
SAMEA6862928
SAMEA6862953
SAMEA6862982
SAMEA6862983
SAMEA6862984
SAMEA6862985
SAMEA6862922
SAMEA6862923
SAMEA6862924
SAMEA6862925
SAMEA6862926
SAMEA6862958
SAMEA6862976
SAMEA6862959
SAMEA6862916
SAMEA6862917
SAMEA6862919
SAMEA6862920
SAMEA6862921
SAMN07678141
SAMEA6862938
SAMEA6862974
SAMEA6862971
SAMEA6862972
SAMEA6862973
SAMN12565078
SAMN12565079
SAMEA6862962
SAMEA6862963
SAMEA6862964

SAMEA6862965
SAMEA6862966
SAMEA6862967
SAMEA6862977
SAMEA6862968
SAMEA6862969
SAMEA6862970
SAMEA6862939
SAMEA6862940
SAMEA6862941
SAMEA6862981
SAMN14210381
SAMN14210380
SAMN14422718
SAMEA6106878
SAMEA6862915
SAMEA6862942
SAMN12565072
SAMEA6862927
SAMEA6862954
SAMEA6862975
SAMEA6862986
SAMEA6862943
SAMEA6862944
SAMEA6862945
SAMEA6862946
SAMEA6862947
SAMN14210375
SAMN14210374
SAMN15232436
SAMN15232437
SAMN15232438
SAMEA6853305
SAMEA6862960
SAMEA6862961
SAMN14210379
SAMN14210378
SAMEA6862948
SAMEA6862949
SAMEA6862950
SAMEA6862951
SAMN14210376
SAMN14210377
SAMN14820580

SAMN14820581
SAMN14820582
SAMN14820583
SAMN14820584
SAMN14820585
SAMEA6862952
SAMN12565080
SAMN12565081
SAMEA6862955
SAMEA6862956
SAMN15650959
SAMN15650961
SAMN15650963
SAMN15650965
SAMN15650967
SAMN15650969
SAMN14279123
SAMEA7198602
SAMEA7198603
SAMEA7198604
SAMEA7198605
SAMEA7198606
SAMEA7198607
SAMEA7198608
SAMEA7198609
SAMEA7198610
SAMEA7198611
SAMEA7198612
SAMEA7297163
SAMEA7297164
SAMEA7297165
SAMEA7297166
SAMEA7297167
SAMEA7297169
SAMEA7297170
SAMN13269014
SAMN13269015
SAMN13269016
SAMN13269017
SAMN13269018
SAMN13269019
SAMN13269020
SAMN13269021
SAMN13269022

SAMN13269023
SAMN13269024
SAMN13269025
SAMN13269026
SAMN13269027
SAMN13269028
SAMN13269029
SAMN13269030
SAMN13269031
SAMN13269032
SAMN13269033
SAMN13269034
SAMN13269035
SAMN13269036
SAMN13269037
SAMN13269038
SAMN13269039
SAMN13269040
SAMN14478636
SAMEA5857935
SAMEA5857938
SAMEA5857941
SAMEA5857950
SAMN02953603
SAMN15625691
SAMN15625692
SAMN15625693
SAMN15625694
SAMN15625695
SAMN15625696
SAMN15625697
SAMN15625698
SAMN15625699
SAMN15625700
SAMN15625701
SAMN15625702
SAMN15625703
SAMN15625704
SAMN15625705
SAMN15625706
SAMN15625707
SAMN15625708
SAMN15625709
SAMN15625710

SAMN15625711
SAMN15625712
SAMN15625713
SAMN15625714
SAMN15625715
SAMN15625716
SAMN15625717
SAMN15625718
SAMN15625719
SAMN15625720
SAMN15625721
SAMN15625722
SAMN15625723
SAMN15625724
SAMN15625725
SAMN15625726
SAMN15625727
SAMN15625728
SAMN15625729
SAMN15625730
SAMN15625731
SAMN15625732
SAMN15625733
SAMN15625734
SAMN15625735
SAMN15625736
SAMN15625737
SAMN15625738
SAMN15625739
SAMN15625740
SAMN15625741
SAMN15625742
SAMN15625743
SAMN15625744
SAMN15625745
SAMN15625746
SAMN15625747
SAMN15625748
SAMN15625749
SAMN15625750
SAMN15625751
SAMN15625752
SAMN15625753
SAMN15625754

SAMN15625755
SAMN15625756
SAMN15625757
SAMN15625758
SAMN15625759
SAMN15625760
SAMN15625761
SAMN15625762
SAMN15625763
SAMN15625764
SAMN15625765
SAMN15625766
SAMN15625767
SAMN15625768
SAMN15625769
SAMN15625770
SAMN15625771
SAMN15625772
SAMN15625773
SAMN15625774
SAMN15625775
SAMN15625776
SAMN15625777
SAMN15625778
SAMN15625779
SAMN15625780
SAMN15625781
SAMN15625782
SAMN15625783
SAMN15625784
SAMN15625785
SAMN15625786
SAMN15625787
SAMN15625788
SAMN15625789
SAMN15625790
SAMN15625791
SAMN15625792
SAMN15625793
SAMN15625794
SAMN15625795
SAMN15625796
SAMN15625797
SAMN15625798

SAMN15625799
SAMN15625800
SAMN15625801
SAMN15625802
SAMN15625803
SAMN15625804
SAMN15625805
SAMN15625806
SAMN15625807
SAMN15625808
SAMN15625809
SAMN15625810
SAMN15625811
SAMN15625812
SAMN15625813
SAMN15625814
SAMN15625815
SAMN15625816
SAMN15625817
SAMN15625818
SAMN15625819
SAMN15625820
SAMN15625821
SAMN15625822
SAMN15625823
SAMN15625824
SAMN15625825
SAMN15625826
SAMN15625827
SAMN15625828
SAMN15625829
SAMN15625830
SAMN15625831
SAMN15625832
SAMN15625833
SAMN15625834
SAMN15625835
SAMN15625836
SAMN15625837
SAMN15625838
SAMN15625839
SAMN15625840
SAMN15625841
SAMN15625842

SAMN15625843
SAMN15625844
SAMN15625845
SAMN15625846
SAMN15625847
SAMN15625848
SAMN15625849
SAMN15625850
SAMN15625851
SAMN15625852
SAMN15625853
SAMN15625854
SAMN15625855
SAMN15625856
SAMN15625857
SAMN15625858
SAMN15625859
SAMN15625860
SAMN15625861
SAMN15625862
SAMN15625863
SAMN15625864
SAMN15625865
SAMN15625866
SAMN15625867
SAMN15625868
SAMN15625869
SAMN15625870
SAMN15625871
SAMN15625872
SAMN15625873
SAMN15625874
SAMN15625875
SAMN15625876
SAMN15625877
SAMN15625878
SAMN15625879
SAMN15625880
SAMN15625881
SAMN15625882
SAMN15625883
SAMN15625884
SAMN15625885
SAMN15625886

SAMN15625887
SAMN15625888
SAMN15625889
SAMN15625890
SAMN15625891
SAMN15625892
SAMN15625893
SAMN15625894
SAMN15625895
SAMN15625896
SAMN15625897
SAMN15625898
SAMN15625899
SAMN15625900
SAMN15625901
SAMN15625902
SAMN15625903
SAMN15625904
SAMN15625905
SAMN15625906
SAMN15625907
SAMN15625908
SAMN15625909
SAMN15625910
SAMN15625911
SAMN15625912
SAMN15625913
SAMN15625914
SAMN15625915
SAMN15625916
SAMN15625917
SAMN15625918
SAMN15625919
SAMN15625920
SAMN15625921
SAMN15625922
SAMN15625923
SAMN15625924
SAMN15625925
SAMN15625926
SAMN15625927
SAMN15625928
SAMN15625929
SAMN15625930

SAMN15625931
SAMN15625932
SAMN15625933
SAMN15625934
SAMN15625935
SAMN15625936
SAMN15625937
SAMN15625938
SAMN15625939
SAMN15625940
SAMN15625941
SAMN15625942
SAMN15625943
SAMN15625944
SAMN15625945
SAMN15625946
SAMN15625947
SAMN15625948
SAMN15625949
SAMN15625950
SAMN15625951
SAMN15625952
SAMN15625953
SAMN15625954
SAMN15625955
SAMN15625956
SAMN15625957
SAMN15625958
SAMN15625959
SAMN15625960
SAMN15625961
SAMN15625962
SAMN15625963
SAMN15625964
SAMN15625965
SAMN15625966
SAMN15625967
SAMN15625968
SAMN15625969
SAMN15625970
SAMN15625971
SAMN15625972
SAMN15625973
SAMN15625974

SAMN15625975
SAMN15625976
SAMN15625977
SAMN15625978
SAMN15625979
SAMN15625980
SAMN15625981
SAMN15625982
SAMN15625983
SAMN15625984
SAMN15625985
SAMN15625986
SAMN15625987
SAMN15625988
SAMN15625989
SAMN15625990
SAMN15625991
SAMN15625992
SAMN15625993
SAMN15625994
SAMN15625995
SAMN15625996
SAMN15625997
SAMN15625998
SAMN15625999
SAMN15626000
SAMN15626001
SAMN15626002
SAMN15626003
SAMN15626004
SAMN15626005
SAMN15626006
SAMN15626007
SAMN15626008
SAMN15626009
SAMN15626010
SAMN15626011
SAMN15626012
SAMN15626013
SAMN15626014
SAMN15626015
SAMN15626016
SAMN15626017
SAMN15626018

SAMN15626019
SAMN15626020
SAMN15626021
SAMN15626022
SAMN15626023
SAMN15626024
SAMN15626025
SAMN15626026
SAMN15626027
SAMN15626028
SAMN15626029
SAMN15626030
SAMN15626031
SAMN15626032
SAMN15626033
SAMN15626034
SAMN15626035
SAMN15626036
SAMN15626037
SAMN15626038
SAMN15626039
SAMN15626040
SAMN15626041
SAMN15626042
SAMN15626043
SAMN15626044
SAMN15626045
SAMN15626046
SAMN15626047
SAMN15626048
SAMN15626049
SAMN15626050
SAMN15626051
SAMN15626052
SAMN15626053
SAMN15626054
SAMN15626055
SAMN15626056
SAMN15626057
SAMN15626058
SAMN15626059
SAMN15626060
SAMN15626061
SAMN15626062

SAMN15626063
SAMN15626064
SAMN15626065
SAMN15626066
SAMN15626067
SAMN15626068
SAMN15626069
SAMN15626070
SAMN15626071
SAMN15626072
SAMN15626073
SAMN15626074
SAMN15626075
SAMN15626076
SAMN15626077
SAMN16976490
SAMN16981799
SAMN15928267
SAMN17045980
SAMN17045981
SAMN17045982
SAMN17045983
SAMN17045984
SAMN17045985
SAMN17045986
SAMN17045987
SAMN17045988
SAMN17045989
SAMN17045990
SAMN17045991
SAMN17045992
SAMN17045993
SAMN17045994
SAMN17045995
SAMN17045996
SAMN17045997
SAMN17045998
SAMN17045999
SAMN17046000
SAMN17046001
SAMN17046002
SAMN17046003
SAMN17046004
SAMN17046005

SAMN17046006
SAMN17046007
SAMN17046008
SAMN17046009
SAMN17046010
SAMN17046011
SAMN17046012
SAMN17046013
SAMN17046014
SAMN17046015
SAMN17046016
SAMN17046017
SAMN17046018
SAMN17046019
SAMN17046020
SAMN17046021
SAMN17046022
SAMN17046023
SAMN17046024
SAMN17046025
SAMN17046026
SAMN17046027
SAMN17046028
SAMN17046029
SAMN17046030
SAMN17046031
SAMN17046032
SAMN17046033
SAMN17046034
SAMN17046035
SAMN17046036
SAMN17046037
SAMN17046038
SAMN17046039
SAMN17046040
SAMN17046041
SAMN17046042
SAMN17046043
SAMN17046044
SAMN17046045
SAMN17046046
SAMN17046047
SAMN17046048
SAMN17046049

SAMN17046050
SAMN17046051
SAMN17046052
SAMN17046053
SAMN17046054
SAMN17046055
SAMN17046056
SAMN17046057
SAMN17046058
SAMN17046059
SAMN17046060
SAMN17046061
SAMN17046062
SAMN17046063
SAMN17046064
SAMN17046065
SAMN17046066
SAMN17046067
SAMN17046068
SAMN17046069
SAMN17046070
SAMN17046071
SAMN17046072
SAMN24256746
SAMN24256747
SAMN24256748
SAMN24256749
SAMN24256750
SAMN24256751
SAMN24256752
SAMN24256753
SAMN24256754
SAMN24256755
SAMN24256756
SAMN24256757
SAMN24256758
SAMN24256759
SAMN24256760
SAMN24256761
SAMN24256762
SAMN24256763
SAMN24256764
SAMN24256765
SAMN24256766

SAMN24256767
SAMN24256768
SAMN24256769
SAMN24256770
SAMN24256771
SAMN24256772
SAMN24256773
SAMN24256774
SAMN24256775
SAMN24256776
SAMN24256777
SAMEA104139329
SAMEA104283460
SAMEA104283461
SAMEA104283462
SAMEA104283463
SAMEA104283464
SAMEA104283465
SAMEA104283466
SAMEA104283467
SAMEA104283468
SAMEA104283469
SAMEA104283470
SAMEA104283471
SAMEA104283472
SAMEA104283473
SAMEA104363589
SAMEA104363593
SAMEA104363604
SAMEA104363610
SAMEA104363614
SAMEA47266168
SAMEA4823359
SAMEA4848668
SAMEA5204132
SAMEA5204133
SAMEA5204134
SAMEA5204135
SAMEA5205212
SAMEA5205213
SAMEA5205214
SAMEA5209170
SAMEA5209171
SAMEA5209172

SAMEA5209173
SAMEA5209174
SAMEA5209175
SAMEA5209176
SAMEA5209177
SAMEA5209178
SAMEA5209179
SAMEA5209180
SAMEA5209181
SAMEA5209182
SAMEA5209183
SAMEA5209184
SAMEA5209185
SAMEA5209186
SAMEA5209187
SAMEA5209188
SAMEA5209189
SAMEA5209190
SAMEA5209191
SAMEA5209192
SAMEA5209193
SAMEA5209194
SAMEA5209195
SAMEA5209196
SAMEA5209197
SAMEA5209198
SAMEA5209199
SAMEA5209200
SAMEA5209201
SAMEA5209202
SAMEA5209203
SAMEA5857937
SAMEA5857945
SAMEA6246836
SAMEA6246837
SAMEA6246838
SAMEA6246839
SAMEA6246840
SAMEA6246841
SAMEA6246842
SAMEA6246843
SAMEA6246844
SAMEA6246845
SAMEA6246846

SAMEA6246847
SAMEA6246848
SAMEA6246849
SAMEA6246850
SAMEA6246851
SAMEA6246852
SAMEA6246853
SAMEA6246854
SAMEA6246855
SAMEA6246856
SAMEA6246857
SAMEA6246858
SAMEA8157152
SAMEA8157153
SAMEA8157154
SAMEA8157155
SAMEA8157156
SAMEA8157157
SAMEA8157158
SAMEA8157159
SAMEA8157160
SAMEA8157161
SAMEA8157162
SAMEA8157163
SAMEA8157164
SAMEA8157165
SAMEA8157166
SAMEA8157167
SAMEA8157168
SAMEA8157169
SAMEA8157170
SAMEA8157171
SAMEA8157172
SAMEA8157173
SAMEA8157174
SAMEA8157175
SAMEA8157176
SAMEA8157177
SAMEA8157178
SAMEA8157179
SAMEA8157180
SAMEA8157181
SAMEA8157182
SAMEA8157183

SAMEA8157184
SAMEA8157185
SAMEA8157186
SAMEA8157187
SAMEA8157188
SAMEA8157189
SAMEA8157190
SAMEA8157191
SAMEA8157192
SAMEA8230809
SAMEA8230810
SAMEA8230811
SAMEA8230812
SAMEA8230813
SAMEA8230814
SAMEA8230815
SAMEA8230816
SAMEA8230817
SAMEA8230818
SAMEA8230819
SAMN17138390
SAMN17192040
SAMN17192041
SAMN17192042
SAMN17192043
SAMN17192044
SAMN17192045
SAMN17192046
SAMN17192047
SAMN17192048
SAMN17192049
SAMN17192050
SAMN17192051
SAMN17192052
SAMN17192053
SAMN17192054
SAMN17192055
SAMN17192056
SAMN17192057
SAMN17192058
SAMN17192059
SAMN17192060
SAMN17192061
SAMN17192062

SAMN17192063
SAMN17192064
SAMN17192065
SAMN17192066
SAMN17192067
SAMN17192068
SAMN17192069
SAMN17192070
SAMN17192071
SAMN17192072
SAMN17192073
SAMN17192074
SAMN17192075
SAMN17192076
SAMN17192077
SAMN17192078
SAMN17192079
SAMN17192080
SAMN17192081
SAMN17192082
SAMN17192083
SAMN17192084
SAMN17192085
SAMN17192086
SAMN17192087
SAMN17192088
SAMN17192089
SAMN17192090
SAMN17192091
SAMN17192092
SAMN17192093
SAMN17192094
SAMN17192095
SAMN17192096
SAMN17192097
SAMN17192098
SAMN17192099
SAMN17192100
SAMN17192101
SAMN17192102
SAMN17192103
SAMN17192104
SAMN17192105
SAMN17192106

SAMN17192107
SAMN17192108
SAMN17192109
SAMN17192110
SAMN17192111
SAMN17192112
SAMN17192113
SAMN17192114
SAMN17192115
SAMN17192116
SAMN17192117
SAMN17192118
SAMN17192119
SAMN17192120
SAMN17192121
SAMN17192122
SAMN17192123
SAMN17192124
SAMN17192125
SAMN17192126
SAMN17192127
SAMN17192128
SAMN17192129
SAMN17192130
SAMN17192131
SAMN12574852
SAMN17983068
SAMEA104090975
SAMEA104090976
SAMEA104090977
SAMEA104090978
SAMEA104090979
SAMEA104090980
SAMEA104090981
SAMEA104090982
SAMEA104090983
SAMEA104090984
SAMEA104090985
SAMEA104090986
SAMEA104090987
SAMEA104090988
SAMEA104090989
SAMEA104090990
SAMEA104090991

SAMEA104090992
SAMEA104090993
SAMEA104090994
SAMEA104090995
SAMEA104090996
SAMEA104090997
SAMEA104090998
SAMEA104090999
SAMEA104091000
SAMEA104091001
SAMEA104091002
SAMEA104091003
SAMEA104091004
SAMEA104091005
SAMEA104091006
SAMEA104091007
SAMEA104091008
SAMEA104091009
SAMEA104091010
SAMEA104091011
SAMEA104091012
SAMEA104091013
SAMEA104091014
SAMEA104091015
SAMEA104091016
SAMEA104091017
SAMEA104091018
SAMEA104091019
SAMEA104091020
SAMEA104091021
SAMEA104091022
SAMEA104091023
SAMEA104091024
SAMEA104091025
SAMEA104091026
SAMEA104091027
SAMEA104091028
SAMEA104091029
SAMEA104091030
SAMEA104091031
SAMEA104091032
SAMEA104091033
SAMEA104091034
SAMEA104091035

SAMEA104091036
SAMEA104091037
SAMEA104091038
SAMEA104091039
SAMEA104091040
SAMEA104091041
SAMEA104091042
SAMEA104091043
SAMEA104091044
SAMEA104091045
SAMEA104091046
SAMEA104091047
SAMEA104091048
SAMEA104091049
SAMEA104091050
SAMEA104091051
SAMEA104091052
SAMEA104091053
SAMEA104091054
SAMEA104091055
SAMEA104091056
SAMEA104091057
SAMEA104091058
SAMEA104091059
SAMEA104091060
SAMEA104091061
SAMEA104091062
SAMEA104091063
SAMEA104091064
SAMEA104091065
SAMEA104091066
SAMEA104091067
SAMEA104091068
SAMEA104091069
SAMEA104091070
SAMEA104091071
SAMEA4853016
SAMEA4853017
SAMEA4853018
SAMEA4853019
SAMEA4853020
SAMEA4853021
SAMEA4853022
SAMEA4853023

SAMEA4853024
SAMEA4853025
SAMEA4853026
SAMEA4853027
SAMEA4853028
SAMEA4853029
SAMEA4853030
SAMEA4853031
SAMEA4853032
SAMEA4853033
SAMEA4853034
SAMEA4853035
SAMEA4853036
SAMEA4853037
SAMEA4853038
SAMEA4853039
SAMEA4853040
SAMEA4853041
SAMEA4853042
SAMEA4853043
SAMEA4853044
SAMEA4853045
SAMEA4853046
SAMEA4853047
SAMEA4853048
SAMEA4853049
SAMEA4853050
SAMEA4853051
SAMEA4853052
SAMEA4853053
SAMEA4853054
SAMEA4853055
SAMEA4853056
SAMEA4853057
SAMEA4853058
SAMEA4853059
SAMEA4853060
SAMEA4853061
SAMEA4853062
SAMEA4853063
SAMEA4853064
SAMEA4853065
SAMEA4853066
SAMEA4853067

SAMEA4853068
SAMEA4853069
SAMEA4853070
SAMEA4853071
SAMEA4853072
SAMEA4853073
SAMEA4853074
SAMEA4853075
SAMEA4853076
SAMEA4853077
SAMEA4853078
SAMEA4853079
SAMEA4853080
SAMEA4853081
SAMEA4853082
SAMEA4853083
SAMEA4853084
SAMEA4853085
SAMEA4853086
SAMEA4853087
SAMEA4853088
SAMEA4853089
SAMEA4853090
SAMEA5883546
SAMEA5883547
SAMEA5883548
SAMEA5883549
SAMEA7038685
SAMEA7038686
SAMEA7038687
SAMEA7038688
SAMEA7038689
SAMEA7038690
SAMEA7038691
SAMEA7038692
SAMEA7038693
SAMEA7038694
SAMEA7038695
SAMEA7038696
SAMEA7038697
SAMEA7038698
SAMEA7038699
SAMEA7038700
SAMEA7038701

SAMEA7038702
SAMEA7038703
SAMEA7038704
SAMEA7038705
SAMEA7038706
SAMEA7038707
SAMEA7038708
SAMEA7038709
SAMEA7038710
SAMEA7038711
SAMEA7038712
SAMEA7038713
SAMEA7038714
SAMEA7038715
SAMEA7038716
SAMEA7038717
SAMEA7038718
SAMEA7038719
SAMEA7038720
SAMEA7038721
SAMEA7038722
SAMEA7091403
SAMN02921310
SAMN02921311
SAMN02921312
SAMN02921313
SAMN02921314
SAMN02921315
SAMN02921316
SAMN02921317
SAMN02921318
SAMN02921319
SAMN02921320
SAMN02921321
SAMN02921322
SAMN03168393
SAMN03168394
SAMN03168395
SAMN03168396
SAMN03168397
SAMN03168398
SAMN03168399
SAMN03168400
SAMN03366710

SAMN03366711
SAMN03366712
SAMN04209256
SAMN05439959
SAMN05791668
SAMN06167395
SAMN06167396
SAMN06219538
SAMN06219539
SAMN08005341
SAMN08005342
SAMN09708541
SAMN10174929
SAMN10174938
SAMN10174939
SAMN10174940
SAMN10174943
SAMN10174944
SAMN10174946
SAMN10174953
SAMN10174954
SAMN10174955
SAMN10180428
SAMN10180429
SAMN10180430
SAMN10180431
SAMN10246085
SAMN10246086
SAMN10246087
SAMN10246088
SAMN10246089
SAMN10246090
SAMN10246091
SAMN10246092
SAMN10246093
SAMN10246094
SAMN10246095
SAMN10246096
SAMN10662571
SAMN10662572
SAMN10662573
SAMN10662574
SAMN10662575
SAMN10662576

SAMN10662577
SAMN10662578
SAMN10662579
SAMN10662580
SAMN10662581
SAMN10662582
SAMN10662583
SAMN10662584
SAMN10662585
SAMN10662586
SAMN10662587
SAMN10662588
SAMN10662589
SAMN10662590
SAMN10662591
SAMN10741007
SAMN10741008
SAMN10741009
SAMN11407875
SAMN11407876
SAMN11407877
SAMN11407878
SAMN11407879
SAMN11407880
SAMN11787846
SAMN11787847
SAMN11787848
SAMN11787849
SAMN11787850
SAMN12565073
SAMN12565074
SAMN12565085
SAMN17023496
SAMN17023497
SAMN17023498
SAMN17023499
SAMN17023500
SAMN17023501
SAMN17023502
SAMN17023503
SAMN17023504
SAMN17023505
SAMN17023506
SAMN17023507

SAMN17023514
SAMN17023515
SAMN17023516
SAMN17023517
SAMN17023518
SAMN17023519
SAMN17023520
SAMN17023521
SAMN19522949
SAMN19522963
SAMEA8797069
SAMEA8797070
SAMEA8797071
SAMEA8797072
SAMEA8797073
SAMEA8797074
SAMEA8797075
SAMEA8797076
SAMEA8797077
SAMEA8797078
SAMEA8797079
SAMEA8797080
SAMEA8797081
SAMEA8797082
SAMEA8797083
SAMEA8797084
SAMEA8797086
SAMEA8797087
SAMEA8797088
SAMEA8797089
SAMEA8797090
SAMEA8797091
SAMEA8797092
SAMEA8797093
SAMEA8797094
SAMEA8797095
SAMEA8797096
SAMN17023483
SAMN17023484
SAMN17023485
SAMN17023486
SAMN17023487
SAMN17023488
SAMN17023489

SAMN17023490
SAMN17023491
SAMN17023492
SAMN17023493
SAMN17023494
SAMN17023495
SAMN17023508
SAMN17023509
SAMN17023510
SAMN17023511
SAMN17023512
SAMN17023513
SAMN18937658
SAMN19522718
SAMN19522719
SAMN19522720
SAMN19522721
SAMN19522722
SAMN19522723
SAMN19522724
SAMN19522725
SAMN19522726
SAMN19522727
SAMN19522728
SAMN19522729
SAMN19522730
SAMN19522731
SAMN19522732
SAMN19522733
SAMN19522734
SAMN19522735
SAMN19522736
SAMN19522737
SAMN19522738
SAMN19522739
SAMN19522740
SAMN19522741
SAMN19522742
SAMN19522743
SAMN19522744
SAMN19522745
SAMN19522746
SAMN19522747
SAMN19522748

SAMN19522749
SAMN19522750
SAMN19522751
SAMN19522752
SAMN19522753
SAMN19522754
SAMN19522755
SAMN19522756
SAMN19522757
SAMN19522758
SAMN19522759
SAMN19522760
SAMN19522761
SAMN19522762
SAMN19522763
SAMN19522764
SAMN19522765
SAMN19522766
SAMN19522767
SAMN19522768
SAMN19522769
SAMN19522770
SAMN19522771
SAMN19522772
SAMN19522773
SAMN19522774
SAMN19522775
SAMN19522776
SAMN19522777
SAMN19522778
SAMN19522779
SAMN19522780
SAMN19522781
SAMN19522782
SAMN19522783
SAMN19522784
SAMN19522785
SAMN19522786
SAMN19522787
SAMN19522788
SAMN19522789
SAMN19522790
SAMN19522791
SAMN19522792

SAMN19522793
SAMN19522794
SAMN19522795
SAMN19522796
SAMN19522797
SAMN19522798
SAMN19522799
SAMN19522800
SAMN19522801
SAMN19522802
SAMN19522803
SAMN19522804
SAMN19522805
SAMN19522806
SAMN19522807
SAMN19522808
SAMN19522809
SAMN19522810
SAMN19522811
SAMN19522812
SAMN19522813
SAMN19522814
SAMN19522815
SAMN19522816
SAMN19522817
SAMN19522818
SAMN19522819
SAMN19522820
SAMN19522821
SAMN19522822
SAMN19522823
SAMN19522824
SAMN19522825
SAMN19522826
SAMN19522827
SAMN19522828
SAMN19522829
SAMN19522830
SAMN19522831
SAMN19522832
SAMN19522833
SAMN19522834
SAMN19522835
SAMN19522836

SAMN19522837
SAMN19522838
SAMN19522839
SAMN19522840
SAMN19522841
SAMN19522842
SAMN19522843
SAMN19522844
SAMN19522845
SAMN19522846
SAMN19522847
SAMN19522848
SAMN19522849
SAMN19522850
SAMN19522851
SAMN19522852
SAMN19522853
SAMN19522854
SAMN19522855
SAMN19522856
SAMN19522857
SAMN19522858
SAMN19522859
SAMN19522860
SAMN19522861
SAMN19522862
SAMN19522863
SAMN19522864
SAMN19522865
SAMN19522866
SAMN19522867
SAMN19522868
SAMN19522869
SAMN19522870
SAMN19522871
SAMN19522872
SAMN19522873
SAMN19522874
SAMN19522875
SAMN19522876
SAMN19522877
SAMN19522878
SAMN19522879
SAMN19522880

SAMN19522881
SAMN19522882
SAMN19522883
SAMN19522884
SAMN19522885
SAMN19522886
SAMN19522887
SAMN19522888
SAMN19522889
SAMN19522890
SAMN19522891
SAMN19522892
SAMN19522893
SAMN19522894
SAMN19522895
SAMN19522896
SAMN19522897
SAMN19522898
SAMN19522899
SAMN19522900
SAMN19522901
SAMN19522902
SAMN19522903
SAMN19522904
SAMN19522905
SAMN19522906
SAMN19522907
SAMN19522908
SAMN19522909
SAMN19522910
SAMN19522911
SAMN19522912
SAMN19522913
SAMN19522914
SAMN19522915
SAMN19522916
SAMN19522917
SAMN19522918
SAMN19522919
SAMN19522920
SAMN19522921
SAMN19522922
SAMN19522923
SAMN19522924

SAMN19522925
SAMN19522926
SAMN19522927
SAMN19522928
SAMN19522929
SAMN19522930
SAMN19522931
SAMN19522932
SAMN19522933
SAMN19522934
SAMN19522935
SAMN19522936
SAMN19522937
SAMN19522938
SAMN19522939
SAMN19522940
SAMN19522941
SAMN19522942
SAMN19522943
SAMN19522944
SAMN19522945
SAMN19522946
SAMN19522947
SAMN19522948
SAMN19522956
SAMN19522957
SAMN19522962
SAMEA7532748
SAMN17849183
SAMEA5875974
SAMEA5875975
SAMEA5875976
SAMEA5875977
SAMEA5875978
SAMEA5875979
SAMEA5875980
SAMEA5875981
SAMEA5875982
SAMEA5875983
SAMEA8008129
SAMEA8008130
SAMEA8008131
SAMEA8008132
SAMEA8008133

SAMEA8008134
SAMEA8008135
SAMEA8008136
SAMEA8008137
SAMEA8008138
SAMEA8008139
SAMEA8008140
SAMEA8008141
SAMEA8008142
SAMEA8008143
SAMEA8008144
SAMEA8008145
SAMEA8008146
SAMEA8008147
SAMEA8008148
SAMEA8008149
SAMEA8008150
SAMEA8797085
SAMN14824620
SAMN14824621
SAMN14824622
SAMN14824623
SAMN17378081
SAMN17378082
SAMN17378083
SAMN17378084
SAMN17378085
SAMN17378092
SAMN17378093
SAMN17378094
SAMN17378095
SAMN17378098
SAMN17378099
SAMN17378104
SAMN17378106
SAMN17378108
SAMN17378131
SAMN17378132
SAMN17378162
SAMN17378165
SAMN17378166
SAMN17378181
SAMN17378182
SAMN17378185

SAMN17378190
SAMN17378192
SAMN17378225
SAMN18253120
SAMN20156216
SAMN20156217
SAMN20156218
SAMN20156219
SAMN20156220
SAMN20833840
SAMN20833841
SAMN20833842
SAMN20833843
SAMN20833844
SAMN20833845
SAMN20833846
SAMN20833847
SAMN20833848
SAMN20833849
SAMN21035041
SAMN21035042
SAMN21035043
SAMN21035044
SAMN21035045
SAMN21035046
SAMN21035047
SAMN21035048
SAMN21035049
SAMN21035050
SAMN21035051
SAMN21035052
SAMN21035053
SAMN21035054
SAMN21035055
SAMN21035056
SAMN21035057
SAMN21035058
SAMN21035059
SAMN21035060
SAMN21035061
SAMN21035062
SAMN21035063
SAMN21035064
SAMN21035065

SAMN21035066
SAMN21035067
SAMN21035068
SAMN21035069
SAMN21035070
SAMN21035071
SAMN21035072
SAMN21035073
SAMN21035074
SAMN21035075
SAMN21035076
SAMN21035077
SAMN21035078
SAMN21035079
SAMN21035080
SAMN21035081
SAMN21035082
SAMN21035083
SAMN21035084
SAMN21035085
SAMN21035086
SAMN21035087
SAMN21035088
SAMN21035089
SAMN21035090
SAMN21035091
SAMN21035092
SAMN21035093
SAMN21035094
SAMN21035095
SAMN21035096
SAMN21035097
SAMN21035098
SAMN21035099
SAMN21035100
SAMN21035101
SAMN21035102
SAMN21035103
SAMN21035104
SAMN21035105
SAMN21035106
SAMN21035107
SAMN21035108
SAMN21035109

SAMN21035110
SAMN21035111
SAMN21035112
SAMN21035113
SAMN21035114
SAMN21035115
SAMN21035116
SAMN21035117
SAMN21035118
SAMN21035119
SAMN21035120
SAMN21035121
SAMN21035122
SAMN21035123
SAMN21035124
SAMN21035125
SAMN21035126
SAMN21035127
SAMN21035128
SAMN21035129
SAMN21035130
SAMN21035131
SAMN21035132
SAMN21035133
SAMN21035134
SAMN21035135
SAMN21035136
SAMN21035137
SAMN21035138
SAMN21035139
SAMN21035140
SAMN21035141
SAMN21035142
SAMN21035143
SAMN21035144
SAMN21035145
SAMN21035146
SAMN21035147
SAMN21035148
SAMN21035149
SAMN21035150
SAMN21035151
SAMN21035152
SAMN21035153

SAMN21035154
SAMN21035155
SAMN21035156
SAMN21035157
SAMN21035158
SAMN21035159
SAMN21035160
SAMN21035161
SAMN21035162
SAMN21035163
SAMN21035164
SAMN21035165
SAMN21035166
SAMN21035167
SAMN21035168
SAMN21035169
SAMN21035170
SAMN21035171
SAMN21035172
SAMN21035173
SAMN21035174
SAMN21035175
SAMN21035176
SAMN21035177
SAMN21035178
SAMN21035179
SAMN21035180
SAMN21035181
SAMN21035182
SAMN21035183
SAMN21035184
SAMN21035185
SAMN21035186
SAMN21035187
SAMN21035188
SAMN21035189
SAMN21035190
SAMN21035191
SAMN21035192
SAMN21035193
SAMN21035194
SAMN21035195
SAMN21035197
SAMN21035198

SAMN21035199
SAMN21035200
SAMN21035201
SAMN21035202
SAMN21035203
SAMN21035204
SAMN21035205
SAMN21035206
SAMN21035207
SAMN21035208
SAMN21035209
SAMN21035210
SAMN21035211
SAMN21035212
SAMN21035213
SAMN21035214
SAMN21035215
SAMN21035216
SAMN21035217
SAMN21035218
SAMN21035219
SAMN21035220
SAMN21035221
SAMN21035222
SAMN21035223
SAMN21035224
SAMN21035225
SAMN21035226
SAMN21035227
SAMN21035228
SAMN21035229
SAMN21035230
SAMN21035231
SAMN21035232
SAMN21035233
SAMN21035234
SAMN21035235
SAMN21035236
SAMN21035237
SAMN21035238
SAMN21035239
SAMN21035240
SAMN21035241
SAMN21035242

SAMN21035243
SAMN21035244
SAMN21035245
SAMN21035246
SAMN21035247
SAMN21035248
SAMN21035249
SAMN21035250
SAMN21035251
SAMN21035252
SAMN21035253
SAMN21035254
SAMN21035255
SAMN21035256
SAMN21035257
SAMN21035258
SAMN21035259
SAMN21035260
SAMN21035261
SAMN21035262
SAMN21035263
SAMN21035264
SAMN21035265
SAMN21035266
SAMN21035267
SAMN21035268
SAMN21035269
SAMN21035270
SAMN21035271
SAMN21035272
SAMN21035273
SAMN21035274
SAMN21035275
SAMN21035276
SAMN21035277
SAMN21035278
SAMN21035279
SAMN21035280
SAMN21035281
SAMN21035282
SAMN21035283
SAMN21035284
SAMN21035285
SAMN21035286

SAMN21035287
SAMN21035288
SAMN21035289
SAMN21035290
SAMN21035291
SAMN21035292
SAMN21035293
SAMN21035294
SAMN21035295
SAMN21035296
SAMN21035297
SAMN21035298
SAMN21035299
SAMN21035300
SAMN21035301
SAMN21035302
SAMN21035303
SAMN21035304
SAMN21035305
SAMN21035306
SAMN21035307
SAMN21035308
SAMN21035309
SAMN21035310
SAMN21035311
SAMN21035312
SAMN21035313
SAMN21035314
SAMN21035315
SAMN21035316
SAMN21035317
SAMN21035318
SAMN21035319
SAMN21035320
SAMN21035321
SAMN21035322
SAMN21035323
SAMN21035324
SAMN21035325
SAMN21035326
SAMN21035327
SAMN21035328
SAMN21035329
SAMN21035330

SAMN21035331
SAMN21035332
SAMN21035333
SAMN21035334
SAMN21035335
SAMN21035336
SAMN21035337
SAMN21035338
SAMN21035339
SAMN21035340
SAMN21035341
SAMN21035342
SAMN21035343
SAMN21035344
SAMN21035345
SAMN21035346
SAMN21035347
SAMN21035348
SAMN21035349
SAMN21035350
SAMN21035351
SAMN21035352
SAMN21035353
SAMN21035354
SAMN21035355
SAMN21035356
SAMN21035357
SAMN21035358
SAMN21035359
SAMN21035360
SAMN21035361
SAMN21035362
SAMN21035363
SAMN21035364
SAMN21035365
SAMN21035366
SAMN21035367
SAMN21035368
SAMN21035369
SAMN21035370
SAMN21035371
SAMN21035372
SAMN21035373
SAMN21035374

SAMN21035375
SAMN21035376
SAMN21035377
SAMN21035378
SAMN21035379
SAMN21035380
SAMN21035381
SAMN21035382
SAMN21035383
SAMN21035384
SAMN21035385
SAMN21035386
SAMN21035387
SAMN21035388
SAMN21035389
SAMN21035390
SAMN21035391
SAMN21035392
SAMN21035393
SAMN21035394
SAMN21035395
SAMN21035396
SAMN21035397
SAMN21035398
SAMN21035399
SAMN21035400
SAMN21035401
SAMN21035402
SAMN21035403
SAMN21035404
SAMN21035405
SAMN21035406
SAMN21035407
SAMN21035408
SAMN21035409
SAMN21035410
SAMN21035411
SAMN21035412
SAMN21035413
SAMN21035414
SAMN21035415
SAMN21035416
SAMN21035417
SAMN21035418

SAMN21035419
SAMN21035420
SAMN21035421
SAMN21035422
SAMN21035423
SAMN21035424
SAMN21035425
SAMN21035426
SAMN21035427
SAMN21035428
SAMN21035429
SAMN21035430
SAMN21035431
SAMN21035432
SAMN21035433
SAMN21035434
SAMN21035435
SAMN21035436
SAMN21035437
SAMN21035438
SAMN21035439
SAMN21035440
SAMN21035441
SAMN21035442
SAMN21035443
SAMN21035444
SAMN21035445
SAMN21035446
SAMN21035447
SAMN21035448
SAMN21035449
SAMN21035450
SAMN21035451
SAMN21035452
SAMN21035453
SAMN21035454
SAMN21035455
SAMN21035456
SAMN21035457
SAMN21035458
SAMN21035459
SAMN21035460
SAMN21035461
SAMN21035462

SAMN21035463
SAMN21035464
SAMN21035465
SAMN21035466
SAMN21035467
SAMN21035468
SAMN21035469
SAMN21035470
SAMN21035471
SAMN21035472
SAMN21035473
SAMN21035474
SAMN21035475
SAMN21035476
SAMN21035477
SAMN21035478
SAMN21035479
SAMN21035480
SAMN21035481
SAMN21035482
SAMN21035483
SAMN21035484
SAMN21035485
SAMN21035487
SAMN21035488
SAMN21035489
SAMN21035490
SAMN21035491
SAMN21035492
SAMN21035493
SAMN21035494
SAMN21035495
SAMN21036177
SAMN21036178
SAMN21036179
SAMN21036180
SAMN21036181
SAMN21036182
SAMN21036183
SAMN21036184
SAMN21036185
SAMN21036186
SAMN21036187
SAMN21036188

SAMN21036189
SAMN21036190
SAMN21036191
SAMN21036192
SAMN21036193
SAMN21036194
SAMN21036195
SAMN21036196
SAMN21036197
SAMN21036198
SAMN21036202
SAMN21036206
SAMN21036207
SAMN21036208
SAMN21036209
SAMN21036210
SAMN21036211
SAMN21036213
SAMN21036214
SAMN21036215
SAMN21036221
SAMN21036222
SAMN21036223
SAMN21036224
SAMN21036225
SAMN21036226
SAMN21036227
SAMN21036228
SAMN21036229
SAMN21036232
SAMN21036233
SAMN21036234
SAMN21036235
SAMN21036236
SAMN21036237
SAMN21036238
SAMN21036239
SAMN21036241
SAMN21036242
SAMN21036248
SAMN21036250
SAMN21036251
SAMN21036252
SAMN21036253

SAMN21036254
SAMN21036255
SAMN21036256
SAMN21036257
SAMN21036258
SAMN21036259
SAMN21036260
SAMN21036261
SAMN21036262
SAMN21036263
SAMN21036264
SAMN21036265
SAMN21036266
SAMN21036267
SAMN21036268
SAMN21036269
SAMN21036270
SAMN21036271
SAMN21036272
SAMN21036273
SAMN21036274
SAMN21036275
SAMN21036281
SAMN21036283
SAMN21036284
SAMN21036285
SAMN21036286
SAMN21036287
SAMN21036288
SAMN21036289
SAMN21036290
SAMN21036291
SAMN21036292
SAMN21036293
SAMN21036294
SAMN21036301
SAMN21036302
SAMN21036303
SAMN21036304
SAMN21036305
SAMN21036306
SAMN21036307
SAMN21036308
SAMN21036309

SAMN21036310
SAMN21036311
SAMN21036312
SAMN21036319
SAMN21036320
SAMN21036321
SAMN21036322
SAMN21036325
SAMN21036328
SAMN21036332
SAMN21036333
SAMN21036334
SAMN21036335
SAMN21036336
SAMN21036337
SAMN21036338
SAMN21036340
SAMN21036344
SAMN21036346
SAMN21036348
SAMN21036349
SAMN21036350
SAMN21036351
SAMN21036352
SAMN21036353
SAMN21036354
SAMN21036355
SAMN21036362
SAMN21036363
SAMN21036364
SAMN21036371
SAMN21036372
SAMN21036373
SAMN21036374
SAMN21036375
SAMN21036376
SAMN21036377
SAMN21036378
SAMN21036379
SAMN21036380
SAMN21036381
SAMN21036382
SAMN21036383
SAMN21036384

SAMN21036385
SAMN21036386
SAMN21036387
SAMN21036394
SAMN21036395
SAMN21036396
SAMN21036397
SAMN21036398
SAMN21036399
SAMN21036400
SAMN21036401
SAMN21036402
SAMN21036403
SAMN21036404
SAMN21036405
SAMN21036406
SAMN21036407
SAMN21036408
SAMN21036409
SAMN21036410
SAMN21036411
SAMN21036412
SAMN21036413
SAMN21036414
SAMN21036415
SAMN21036416
SAMN21036417
SAMN21036418
SAMN21036419
SAMN21036420
SAMN21036421
SAMN21036422
SAMN21036423
SAMN21036424
SAMN21036425
SAMN21036426
SAMN21036427
SAMN21036428
SAMN21036429
SAMN21036430
SAMN21036431
SAMN21036432
SAMN21036433
SAMN21036434

SAMN21036435
SAMN21036436
SAMN21036437
SAMN21036438
SAMN21036439
SAMN21036440
SAMN21036441
SAMN21036442
SAMN21036443
SAMN21036444
SAMN21036445
SAMN21036446
SAMN21036448
SAMN21036454
SAMN21036455
SAMN21036456
SAMN21036457
SAMN21036458
SAMN21036459
SAMN21036460
SAMN21036467
SAMN21036468
SAMN21036469
SAMN21036470
SAMN21036471
SAMN21036472
SAMN21036473
SAMN21036474
SAMN21036475
SAMN21036482
SAMN21036483
SAMN21036484
SAMN21036485
SAMN21036486
SAMN21036489
SAMN21036492
SAMN21036495
SAMN21036496
SAMN21036497
SAMN21036498
SAMN21036499
SAMN21036500
SAMN21036501
SAMN21036502

SAMN21036503
SAMN21036504
SAMN21036505
SAMN21036506
SAMN21036507
SAMN21036509
SAMN21036510
SAMN21036511
SAMN21036512
SAMN21036513
SAMN21036514
SAMN21036515
SAMN21036516
SAMN21036517
SAMN21036519
SAMN21036520
SAMN21036526
SAMN21036534
SAMN21036535
SAMN21036536
SAMN21036199
SAMN21036200
SAMN21036201
SAMN21036203
SAMN21036204
SAMN21036205
SAMN21036212
SAMN21036216
SAMN21036217
SAMN21036218
SAMN21036219
SAMN21036220
SAMN21036243
SAMN21036244
SAMN21036245
SAMN21036246
SAMN21036247
SAMN21036249
SAMN21036276
SAMN21036277
SAMN21036278
SAMN21036279
SAMN21036280
SAMN21036282

SAMN21036295
SAMN21036296
SAMN21036297
SAMN21036298
SAMN21036299
SAMN21036300
SAMN21036313
SAMN21036314
SAMN21036315
SAMN21036316
SAMN21036318
SAMN21036323
SAMN21036324
SAMN21036326
SAMN21036327
SAMN21036329
SAMN21036330
SAMN21036339
SAMN21036341
SAMN21036342
SAMN21036343
SAMN21036345
SAMN21036347
SAMN21036356
SAMN21036357
SAMN21036358
SAMN21036359
SAMN21036360
SAMN21036361
SAMN21036365
SAMN21036366
SAMN21036367
SAMN21036368
SAMN21036369
SAMN21036370
SAMN21036388
SAMN21036389
SAMN21036390
SAMN21036391
SAMN21036392
SAMN21036393
SAMN21036447
SAMN21036449
SAMN21036450

SAMN21036451
SAMN21036452
SAMN21036453
SAMN21036461
SAMN21036462
SAMN21036463
SAMN21036464
SAMN21036465
SAMN21036466
SAMN21036476
SAMN21036477
SAMN21036478
SAMN21036479
SAMN21036480
SAMN21036481
SAMN21036487
SAMN21036488
SAMN21036490
SAMN21036491
SAMN21036493
SAMN21036494
SAMN21036508
SAMN21036518
SAMN21036521
SAMN21036522
SAMN21036523
SAMN21036524
SAMN21036525
SAMN21036528
SAMN21036529
SAMN21036530
SAMN21036531
SAMN21036532
SAMN21036533
SAMN39309472
SAMN39309473
SAMN39309474
SAMN39309475
SAMN39309476
SAMN39309477
SAMN39309478
SAMN39309479
SAMN39309480
SAMN39309481

SAMN39309482
SAMN39309483
SAMN39309484
SAMN39309485
SAMN39309486
SAMN39309487
SAMN39309488
SAMN39309489
SAMN39309490
SAMN39309491
SAMN39309492
SAMN39309493
SAMN39309494
SAMN39309495
SAMN39309496
SAMN39309497
SAMN39309498

ding order with rpoband CNP Weimaraner highlighted at the top.
