# Supplementary material for: Homozygous CNP Mutation and Neurodegeneration in Weimaraners: Myelin Abnormalities and Accumulation of Lipofuscin-like Inclusions
Source: Genes (Basel). 2024 Feb 15;15(2):246. doi: 10.3390/genes15020246 (PMC10888007; doi:10.3390/genes15020246)
Supplement: Supplementary file 1 [file genes-15-00246-s001.zip › Supplementary File S2 - AA sequence human dog align.pdf]

Supplementary File S2: Amino acid sequence alignments of human and canine CNPase isoforms 2 showing the locations of the disease-related variants in the canine (green T42M) and human (blue S82L)

```
CLUSTAL O(1.2.4) multiple sequence alignment


sp|P09543|CN37_HUMAN                MNRGFSRKSHTFLPKIFFRKMSSSGAKDKPELQFPFLQDEDTVATLLECKTLFILRGLPG    60
tr|A0A8I3NGU6|A0A8I3NGU6_CANLF      MNRSFSRKSHAFLPKIFFRKMSSSGAKDKPELQFPFLQDEETVATLQECKTLFILRGLPG    60
                                                                             M
                                                         L
sp|P09543|CN37_HUMAN                SGKSTLARVIVDKYRDGTKMVSADAYKITPGARGAFSEEYKRLDEDLAAYCRRRDIRILV    120
tr|A0A8I3NGU6|A0A8I3NGU6_CANLF      SGKSTLARVIVDRYRDGTKMVSADAYKITPGARGDFSEEYKRLDEDLAAYCR-RDVRVLV    119

sp|P09543|CN37_HUMAN                LDDTNHERERLEQLFEMADQYQYQVVLVEPKTAWRLDCAQLKEKNQWQLSADDLKKLKPG    180
tr|A0A8I3NGU6|A0A8I3NGU6_CANLF      LDDTNHERERLEQLFELADQYQYQVVLVEPKTAWRLDCAQLKEKNQWQLSADELKKLKPG    179

sp|P09543|CN37_HUMAN                LEKDFLPLYFGWFLTKKSSETLRKAGQVFLEELGNHKAFKKELRQFVPGDEPREKMDLVT    240
tr|A0A8I3NGU6|A0A8I3NGU6_CANLF      LEKDFLPLYFGWFLTKKSSESLRKAGQAFLEELGNHKAFKKELRHFVSGDEPREKIELVT    239

sp|P09543|CN37_HUMAN                YFGKRPPGVLHCTTKFCDYGKAPGAEEYAQQDVLKKSYSKAFTLTISALFVTPKTTGARV    300
tr|A0A8I3NGU6|A0A8I3NGU6_CANLF      YFGKRPPGVLHCTTKFCDYGKAAGADEYAQQDVVKKSYCKAFTLTITALFVTPKTAGARV    299

sp|P09543|CN37_HUMAN                ELSEQQLQLWPSDVDKLSPTDNLPRGSRAHITLGCAADVEAVQTGLDLLEILRQEKGGSR    360
tr|A0A8I3NGU6|A0A8I3NGU6_CANLF      ELSEQELPLWPNDVDKLSPSDSLPRGSRAHITLGCAGDVEPVQTGIDLLEIVRQEKGGSR    359

sp|P09543|CN37_HUMAN                GEEVGELSRGKLYSLGNGRWMLTLAKNMEVRAIFTGYYGKGKPVPTQGSRKGGALQSCTI    420
tr|A0A8I3NGU6|A0A8I3NGU6_CANLF      GEEVGELNRGKLYSLGNGRWLLSLAKKLEVRAIFTGYYGKGKPVPTHGSRKGGALQSCTI    419

sp|P09543|CN37_HUMAN                I 421
tr|A0A8I3NGU6|A0A8I3NGU6_CANLF      I 420
```
